# Structural mechanism of bridge RNA-guided recombination

**DOI:** 10.1038/s41586-024-07570-2

**Published:** 2024-06-26

**Authors:** Masahiro Hiraizumi, Nicholas T. Perry, Matthew G. Durrant, Teppei Soma, Naoto Nagahata, Sae Okazaki, Januka S. Athukoralage, Yukari Isayama, James J. Pai, April Pawluk, Silvana Konermann, Keitaro Yamashita, Patrick D. Hsu, Hiroshi Nishimasu

**Affiliations:** 1 Department of Chemistry and Biotechnology, Graduate School of Engineering, The University of Tokyo, Tokyo, Japan; 2 Arc Institute, Palo Alto, CA USA; 3 Department of Bioengineering, University of California, Berkeley, Berkeley, CA USA; 4 San Francisco Graduate Program in Bioengineering, University of California, Berkeley, Berkeley, CA USA; 5 Structural Biology Division, Research Center for Advanced Science and Technology, The University of Tokyo, Tokyo, Japan; 6 Department of Biochemistry, Stanford University School of Medicine, Stanford, CA USA; 7 Center for Computational Biology, University of California, Berkeley, Berkeley, CA USA; 8 Inamori Research Institute for Science, Kyoto, Japan

**Keywords:** Structural biology, Enzyme mechanisms, Transposition, DNA recombination

## Abstract

Insertion sequence (IS) elements are the simplest autonomous transposable elements found in prokaryotic genomes^1^. We recently discovered that IS110 family elements encode a recombinase and a non-coding bridge RNA (bRNA) that confers modular specificity for target DNA and donor DNA through two programmable loops^2^. Here we report the cryo-electron microscopy structures of the IS110 recombinase in complex with its bRNA, target DNA and donor DNA in three different stages of the recombination reaction cycle. The IS110 synaptic complex comprises two recombinase dimers, one of which houses the target-binding loop of the bRNA and binds to target DNA, whereas the other coordinates the bRNA donor-binding loop and donor DNA. We uncovered the formation of a composite RuvC–Tnp active site that spans the two dimers, positioning the catalytic serine residues adjacent to the recombination sites in both target and donor DNA. A comparison of the three structures revealed that (1) the top strands of target and donor DNA are cleaved at the composite active sites to form covalent 5′-phosphoserine intermediates, (2) the cleaved DNA strands are exchanged and religated to create a Holliday junction intermediate, and (3) this intermediate is subsequently resolved by cleavage of the bottom strands. Overall, this study reveals the mechanism by which a bispecific RNA confers target and donor DNA specificity to IS110 recombinases for programmable DNA recombination.

## Abstract (web summary)

Using cryo-electron microscopy, the structural mechanism by which non-coding bridge RNA confers target and donor DNA specificity to IS110 recombinases for programmable DNA recombination is explored.

## Main

Transposable elements are mobile DNA sequences that can move or copy themselves to new locations within genomes. They are widespread throughout all domains of life and have vital roles in shaping genome function and evolution^3^. Transposons typically encode a transposase gene and terminal inverted repeats at both ends of the elements. Using diverse catalytic mechanisms, these transposases recognize the inverted repeats to catalyse the excision and insertion of the transposable element into new target sites in the genome^4^.

**Fig. 1:**
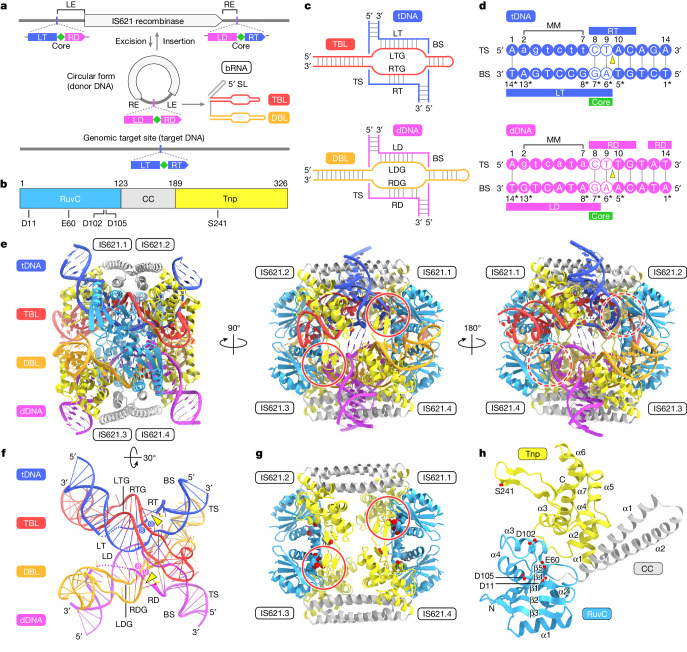
IS621 synaptic complex structure. **a**, Schematic of the IS621 insertion sequence element. LD, left donor; RD, right donor; LE, left end; RE, right end, LT, left target; RT, right target; SL, stem loop. **b**, Domain structure of the IS621 recombinase. CC, coiled-coil domain. **c**, Schematics of bRNA-guided dDNA and tDNA recognition. The 5′ stem loop and the linker region in the bRNA are omitted, as the TBL and DBL in a single synaptic complex are derived from two different bRNA molecules. BS, bottom strand; TS, top strand. **d**, Nucleotide sequences of the bRNA-complementary regions in the tDNA and dDNA. Mismatched (MM) nucleotides introduced to the top strands for the structural analysis are shown as lowercase letters. Bottom strands are indicated by asterisks. **e**,**f**, Structures of the IS621–bRNA–dDNA–tDNA synaptic complex (**e**) and the bRNA–dDNA–tDNA complex (**f**). Disordered regions are indicated by dotted lines, and the CT core dinucleotides (positions 8 and 9) are numbered. In **f**, the S241 residues are shown as stick models. **g**,**h**, Structures of the tetramer (**g**) and monomer (**h**) of the IS621 recombinase. The catalytic residues are shown as space-filling (**g**) and stick (**h**) models. In **h**, the core α-helices and β-strands in each domain are numbered. In **d**,**f**, DNA cleavage sites are indicated by yellow triangles. In **e**,**g**, the active sites with the ordered and disordered S241 residues are indicated by red solid and dashed circles, respectively.

**Extended Data Fig. 1:**
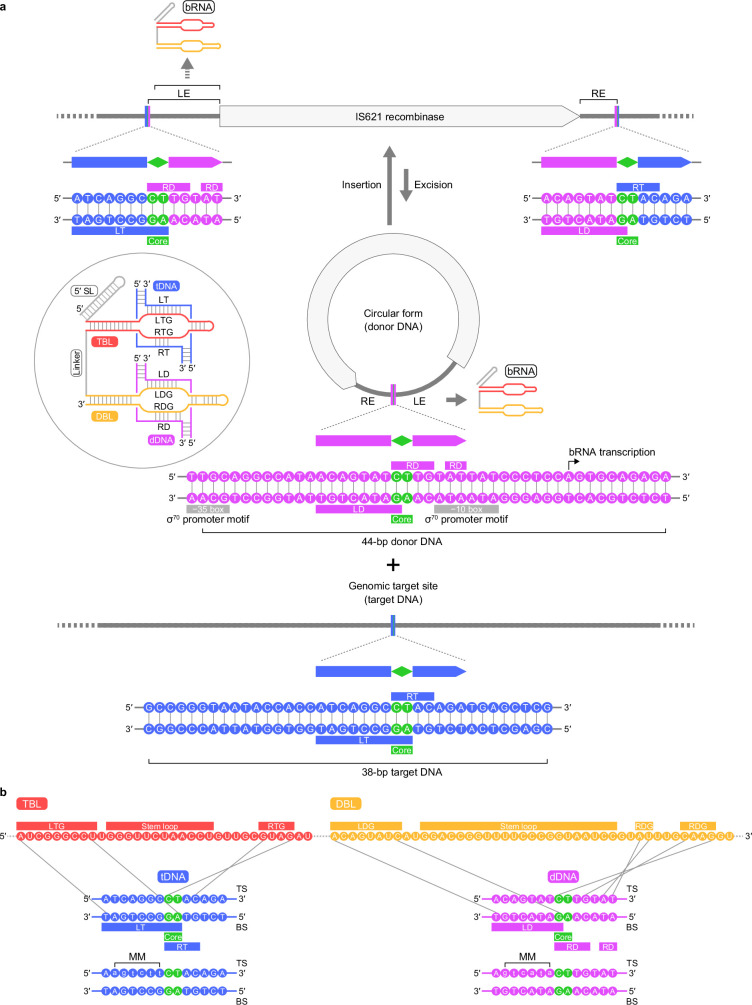
IS621 insertion sequence element. (**a**) Transposition cycle of the IS621 insertion sequence element. The IS621 elements consist of the left end (LE), the recombinase-coding sequence, and the right end (RE), flanked by the CT core dinucleotide sequences at both ends. The transposition cycle of the IS621 elements consists of excision (generation of a circular form) and insertion (recombination between the circular form and genomic target sites) steps. In the excision step, recombination would occur between the 5′ left target (LT)–core–right donor (RD) region and the 3′ left donor (LD)–core–right target (RT) region in the IS621 locus, resulting in the LT–core–RT region in the original genomic site and the LD–core–RD region at the RE–LE junction in the circular form. Importantly, the RE–LE junction contains the reconstituted σ^70^-like promoter sequence, since RE and LE contain a −35 box and a −10 box, respectively^2^. Thus, the bRNA encoded in the LE is expressed downstream of the core sequence at the RE–LE junction in the circular intermediate^2^. However, it remains unclear whether the excision is mediated by the IS621–bRNA complex and, if so, how the IS621–bRNA complex accomplishes both excision and insertion reactions. For our cryo-EM analysis, we used the 44-bp donor DNA (the RE–LE junction with the LD–core–RD sequence in the circular form) and the 38-bp target DNA (the genomic target site with the LT–core–RT sequence) from the natural IS621 element found in *E. coli*, with the indicated minor modifications to assist structural analysis. 5′ SL, 5′ stem loop; TBL, target-binding loop; DBL, donor-binding loop; LTG, left target guide; RTG, right target guide; LDG, left donor guide; RDG, right donor guide; TS, top strand; BS, bottom strand. (**b**) Schematics showing base-pairing between the bRNA and tDNA/dDNA. Mismatched (MM) nucleotides introduced to the top strands for the structural analysis are shown as lower-case letters.

Insertion sequence elements are the simplest autonomous transposable elements found in prokaryotic genomes and are classified into approximately 30 families^1^. Typical insertion sequence elements are excised and then randomly inserted into new genomic loci by the actions of their cognate transposases. By contrast, the IS110 family elements are excised as circular double-stranded DNA intermediates and then inserted into specific DNA target sequences through unknown mechanisms^5–10^ (Fig. 1a and Extended Data Fig. 1a). Given the conservative transposition cycle of IS110 elements, their encoded transposases can be referred to as recombinases, reflecting a closer functional resemblance to site-specific recombinases such as Bxb1 and Cre^11,12^. IS110 family elements consist of a left end, a recombinase-coding sequence and a right end, flanked by the CT (cytosine–thymine) core dinucleotide sequences (Fig. 1a and Extended Data Fig. 1a), and unlike most other insertion sequence elements, they do not encode long terminal inverted repeat sequences, further obscuring their transposition mechanism. Consistent with these unique features of the IS110 elements, the IS110 recombinases differ from other known enzymes and comprise a RuvC-like domain (Pfam PF01548, hereafter referred to as RuvC for simplicity) with a DEDD motif^7^, as well as a Tnp domain (Pfam PF02371) with a conserved serine residue^2^ (Fig. 1b and Supplementary Fig. 1). These sequence features and functional observations indicate that IS110 elements use a distinctive mechanism of action that has, until now, remained elusive.

In our accompanying study^2^, we established that the *Escherichia coli* IS621 element^7^, a member of the IS110 family, reconstitutes a σ^70^-like promoter at the right end–left end junction in its circular intermediate form to express a bRNA from the left end (Fig. 1a and Extended Data Fig. 1a). This bRNA encodes specificity determinants for both donor DNA (dDNA; the IS621 circular form) and target DNA (tDNA; the genomic insertion site) (Fig. 1c and Extended Data Fig. 1a,b). The bRNA target-binding loop (TBL) contains two guide segments: a left target guide (LTG) and a right target guide (RTG), which base pair with the left side of the bottom strand (left target) and the right side of the top strand (right target) of a double-stranded tDNA, respectively (Fig. 1c, Extended Data Fig. 1a,b and Supplementary Fig. 2a). The analogous architecture of the donor-binding loop (DBL; composed of a left donor guide (LDG) and a right donor guide (RDG)) base pairs with the left donor and right donor regions of a double-stranded dDNA in a similar manner. Functionally, the IS621 recombinase associates with the bRNA and catalyses recombination between dDNA and tDNA at a central CT core dinucleotide present in both DNA molecules (Fig. 1a and Extended Data Fig. 1a,b). We demonstrated its broad and modular reprogrammability to desired dDNA and tDNA substrates for genome insertion, inversion and excision, laying the groundwork for a unified mechanism for programmable DNA rearrangements^2^. In this study, we solved the high-resolution cryo-electron microscopy (cryo-EM) structures of the IS621 synaptic complexes in several reaction intermediate states, revealing the bRNA-guided DNA recombination mechanism.

## IS621 synaptic complex structure

**Extended Data Fig. 2:**
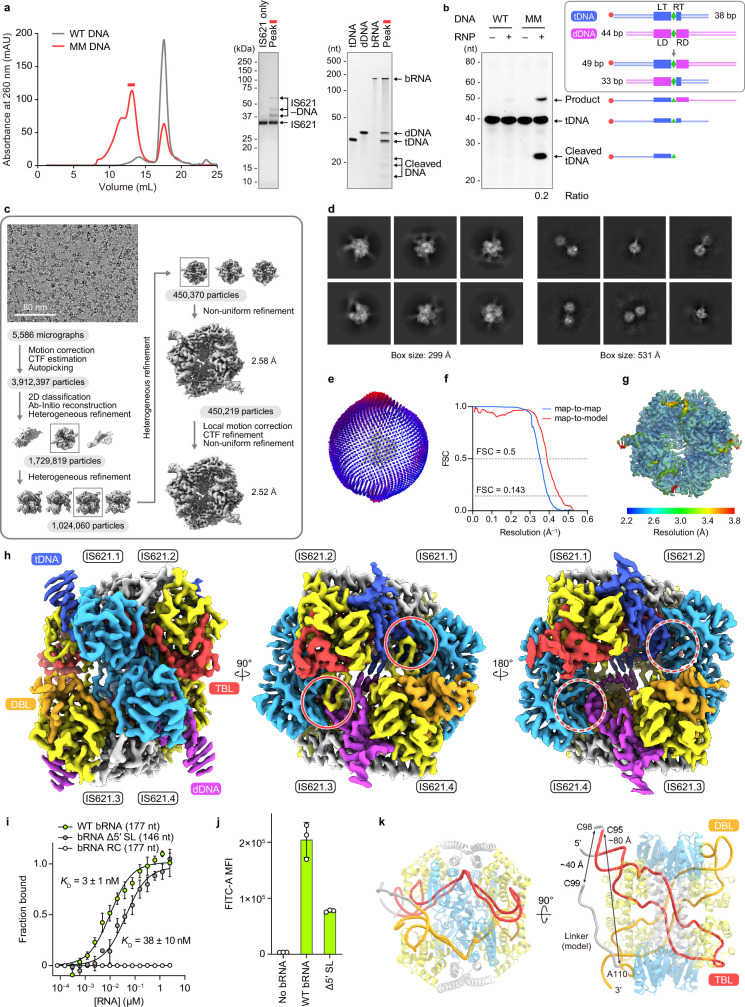
Cryo-EM analysis of the IS621 synaptic complex. (**a**) Preparation of the IS621–bRNA–dDNA–tDNA synaptic complex. The IS621 recombinase, bRNA, and dDNA/tDNA (containing no mismatch (WT) or 6-nt mismatches (MM) at positions 2–7 in dDNA and tDNA) were mixed, and then purified by a Superose 6 Increase 10/300 column. The peak fraction of the synaptic complex with the mismatched DNA (indicated by a red line) was analyzed by SDS-PAGE (10–20%) and TBE–urea PAGE (15%). The proteins and nucleic acids were visualized with CBB and SYBR Gold, respectively. We observed three slower-migrating bands that may correspond to covalent IS621–DNA intermediates during recombination (probably IS621–dDNA, IS621–tDNA, and IS621–dDNA–tDNA). Experiments were repeated at least three times with similar results. (**b**) In vitro DNA recombination experiments. The 38-bp tDNA and 44-bp dDNA substrates (containing no mismatch (WT) or 6-nt mismatches (MM) at positions 2–7 in their top strands) were incubated with the IS621–bRNA complex at 37 °C for 1 h, and then the reaction was analyzed using an 18% TBE–urea gel. The tDNA was labeled with Cy5 at the 5′ end of the top strand. The band intensities of the product and cleaved DNAs were quantified, and the recombination ratios (product DNA / product DNA + cleaved DNA) were calculated. Experiments were repeated at least three times with similar results. (**c**) Single-particle cryo-EM image processing workflow. (**d**) Representative 2D averaged class images. (**e**) Angular distribution of particles in the final reconstruction. (**f**) Fourier shell correlation (FSC) curves. The map-to-map FSC curve was calculated between the two independently refined half-maps after masking (blue line), and the overall resolution was determined by the gold standard FSC = 0.143 criterion. The map-to-model FSC curve was calculated between the refined atomic model and the full map (red line). (**g**, **h**) Cryo-EM density maps, colored according to the local resolution (**g**) and the protein domains (**h**). In (**h**), the active sites with the ordered and disordered S241 residues are indicated by red solid and dashed circles, respectively. (**i**) Effects of 5′ SL deletion on bRNA binding to the IS621 recombinase. Binding of the purified IS621 recombinase to the bRNA, its reverse complement (RC), or the bRNA lacking the 5′ SL (nucleotides A1–U33) (Δ5′ SL) was analyzed using microscale thermophoresis. Data are shown as mean ± SEM for three technical replicates. (**j**) Effects of 5′ SL deletion on IS621-mediated recombination in *E. coli*. Data are shown as mean ± SD for three biological replicates. (**k**) Distance between TBL and DBL. Nucleotides U96–A109 are disordered in the present structure. Modeling of nucleotides U96–A109 (colored grey) suggests that C98 and C99 are ~40-Å apart, indicating that the TBL and DBL in the synaptic complex structure are derived from two different bRNA molecules.

For cryo-EM analysis, we first attempted to reconstitute the synaptic complex comprising the IS621 recombinase, a full-length 177-nucleotide (nt) bRNA, a 44-base pair (bp) dDNA (the right end–left end junction with the left donor–core–right donor sequence in the circular form) and a 38-bp tDNA (the genomic insertion site with the left target–core–right target sequence) from the natural IS621 element found in *E. coli* (Fig. 1b,c, Extended Data Fig. 1a,b and Supplementary Fig. 2a). However, the IS621–bRNA complex did not form a stable synaptic complex with dDNA and tDNA on size-exclusion chromatography (Extended Data Fig. 2a). To facilitate bRNA-mediated DNA targeting, we introduced mismatches into the top strand of the dDNA (6 nt) and tDNA (6 nt) at positions 2–7, and found that these mismatches facilitated synaptic complex formation and in vitro DNA recombination (Fig. 1d and Extended Data Figs. 1a,b and 2a,b). Using these mismatch-containing dDNA and tDNA molecules, we determined the cryo-EM structure of the IS621–bRNA–dDNA–tDNA synaptic complex at 2.5 Å resolution (Extended Data Fig. 2c–h).

The synaptic complex consists of four IS621 recombinase protomers (IS621.1–4) and the TBL and DBL modules of the bRNA, as well as tDNA and dDNA (Fig. 1e–g and Supplementary Video 1). The 5′ stem loop of the bRNA, as well as the linker region between the TBL and DBL, are not visible in the density map (Extended Data Fig. 2h), suggesting their flexibility. Deletion of the 5′ stem loop decreased bRNA binding to the IS621 recombinase (Extended Data Fig. 2i) and slightly reduced IS621-mediated recombination in *E. coli* (Extended Data Fig. 2j), suggesting that the 5′ stem loop does not form specific interactions with the recombinase, but rather enhances the IS621–bRNA interaction, possibly through nonspecific RNA backbone interactions. The 5′ stem loops are structurally conserved among IS110 orthologues, although their sequences are diverse^2^, suggesting their conserved functional roles. In the structure, the TBL 3′ end (C95) and the DBL 5′ end (A110) are approximately 80 Å apart (Extended Data Fig. 2k), suggesting that two different bRNA molecules contribute the TBL and DBL, respectively, to a single synaptic complex. Supporting this notion, we observed dimers of the synaptic complex during 2D classification, in which two synaptic complexes are probably held together by two bRNA molecules (Extended Data Fig. 2d).

IS621.1 and IS621.2 form a dimer that interacts with the TBL and tDNA, while IS621.3 and IS621.4 form a dimer that interacts with the DBL and dDNA (Fig. 1e). Consistent with our functional data showing RNA-guided DNA recognition by the IS621 recombinase^2^, the TBL and DBL base pair with tDNA and dDNA, respectively (Fig. 1f). The tDNA and dDNA molecules bend around the central catalytic site, adopting an X-shaped architecture (Fig. 1e,f). Consistent with the observed recombination adjacent to the CT core sequences^2^, both the tDNA (between tT9 and tA10) and the dDNA (between dT9 and dT10) are cleaved adjacent to the CT core sequences (tC8–tT9 and dC8–dT9), with the conserved serine residues (S241) in IS621.4 and IS621.2 forming covalent 5′-phosphoserine linkages with tDNA tA10 and dDNA dT10, respectively (Fig. 1e,f). As observed for serine recombinases^13^, SDS–PAGE analysis of the synaptic complex revealed slower-migrating bands that probably correspond to covalent IS621–DNA complexes (Extended Data Fig. 2a). We conclude that this structure represents the post-top strand cleavage state of the IS621 synaptic complex before the dDNA and tDNA strands are exchanged.

## IS621 recombinase structure

**Extended Data Fig. 3:**
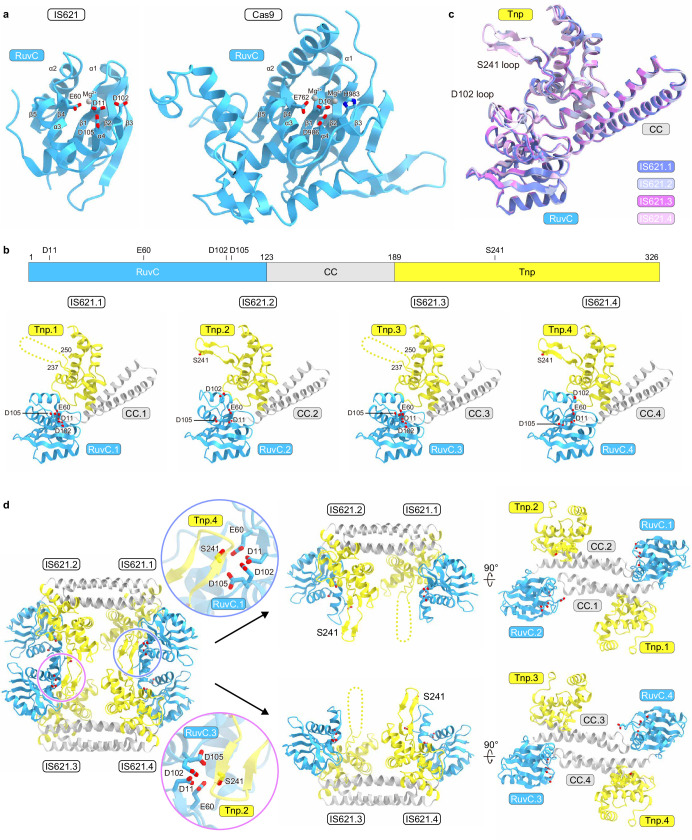
IS621 recombinase structure. (**a**) Structural comparison of the RuvC domains of IS621 and Cas9 (PDB: 7S4X). The catalytic residues are shown as stick models. The core α-helices and β-strands are labeled. The RuvC active site of IS621 plays a role in coordinating a Mg^2+^ ion that stabilizes the 5′-phosphoserine DNA intermediates, thereby facilitating DNA cleavage and religation. In contrast, most RuvC domains, such as that of Cas9, bind two Mg^2+^ ions and catalyse the DNA cleavage reaction (*i.e*., the nucleophilic attack of an activated water molecule on the scissile phosphodiester bond in a substrate DNA). (**b**) Structures of the four IS621 protomers. The disordered S241 loops in IS621.1 and IS621.3 are indicated by dotted lines. (**c**) Superimposition of the RuvC domains in the four IS621 protomers. (**d**) Structure of the IS621 tetramer. The active sites formed by RuvC.1–Tnp.4 and RuvC.3–Tnp.2 are indicated by blue and magenta circles, respectively.

The IS621 recombinase consists of a RuvC domain (residues 1–122), a two-stranded α-helical coiled-coil domain (residues 123–188) and a Tnp domain (residues 189–326) (Fig. 1b,h). The RuvC domain adopts an RNase H fold, comprising a five-stranded β-sheet flanked by four α-helices, with an active site formed by the DEDD motif (D11, E60, D102 and D105) (Fig. 1h). Although the RuvC domain of IS621 shares structural similarity to those of other proteins, such as Cas9, the third residue in the DEDD motif (D102 in IS621) is often replaced in other RuvC domains^14–16^ (Extended Data Fig. 3a). The Tnp domain comprises seven α-helices, with the conserved serine residue S241 located in a loop region between helices α3 and α4 (Fig. 1h). A Dali search^17^ revealed that the Tnp domain lacks detectable structural similarity with any other known proteins. The coiled-coil domain contains two α-helices and mediates the dimerization between IS621.1 and IS621.2 and between IS621.3 and IS621.4 (Fig. 1g,h).

The four IS621 protomers are structurally similar, except for the D102 loops in the RuvC domains and the S241 loops in the Tnp domains (Extended Data Fig. 3b,c). The S241 loops in IS621.2 and IS621.4 are ordered and interact with the D102 loops in IS621.3 and IS621.1, respectively, forming composite active sites that span between the two distinct dimers in the synaptic complex (Fig. 1g and Extended Data Fig. 3d). By contrast, the S241 loops in IS621.1 and IS621.3 are disordered, with the D102 loops in IS621.2 and IS621.4 adopting distinct conformations from those in IS621.1 and IS621.3 (Extended Data Fig. 3b,c). Whereas typical RuvC-like domains do not require other domains for their catalytic activity, the RuvC domains of the IS110 recombinases function together with their Tnp domains, with the third residue in the IS110-specific DEDD motif (D102 in IS621) forming a composite active site with S241 of the IS110-specific Tnp domain. This distinctive arrangement of the catalytic residues probably helps to connect the two dimers in the synaptic complex and prevent DNA cleavage from occurring before synaptic complex formation.

## bRNA architecture

**Fig. 2:**
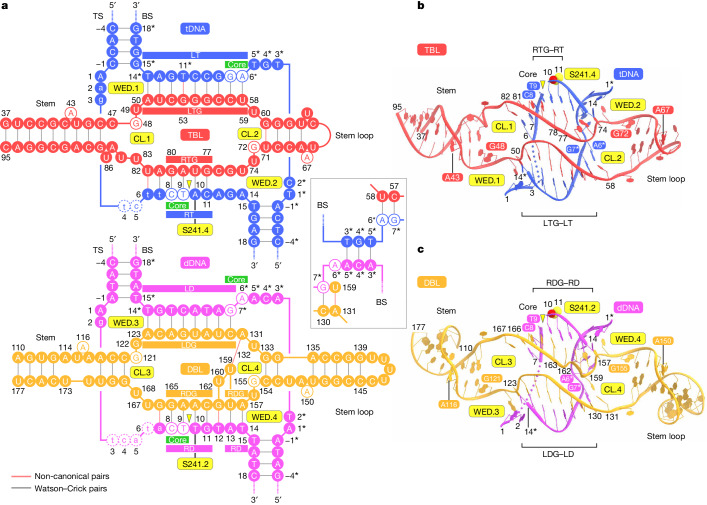
bRNA architecture. **a**, Schematics showing base pairing between the bRNA and tDNA (top) and bRNA and dDNA (bottom). The covalent 5′-phosphoserine–DNA linkages are indicated by grey lines. Non-canonical base pairing is indicated by red lines. Disordered nucleotides are indicated by dashed circles. The 5′ stem loop and the linker region are omitted. CL, catalytic loop; WED, hydrophobic wedge. **b**,**c**, Structures of TBL–tDNA (**b**) and DBL–dDNA (**c**). Disordered regions are indicated by dotted lines. The S241 residues are depicted as space-filling models. In **a**–**c**, DNA cleavage sites are indicated by yellow triangles.

**Extended Data Fig. 4:**
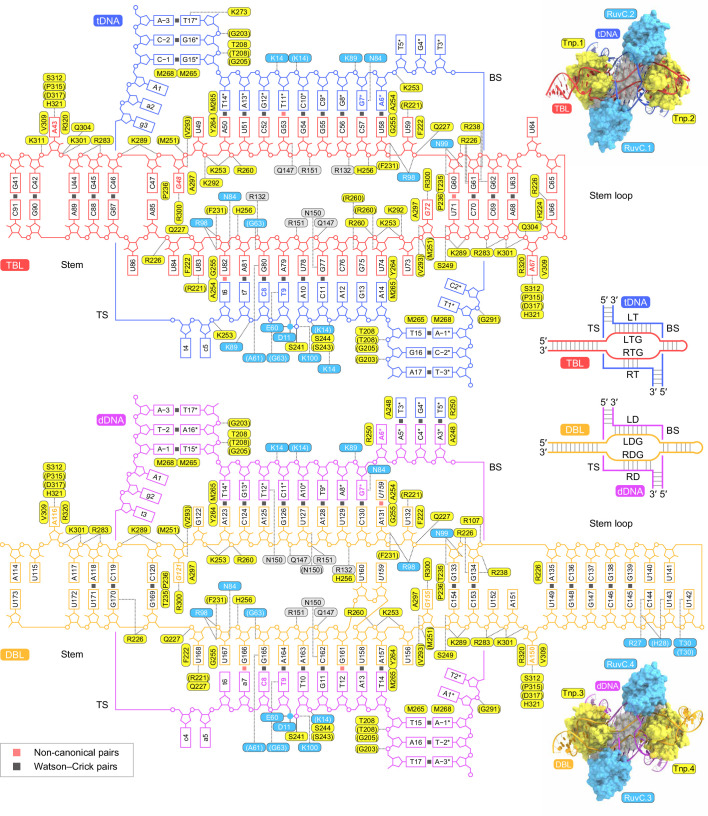
Schematic of interactions between IS621 and nucleic acids. A43/A67/A116/A150 and G48/G72/G121/G155, common structural features in the TBL and DBL, and the core nucleotides (tC8/tT9/tA6*/tG7* and dC8/dT9/dA6*/dG7*) are highlighted in bold. G48/G72/G121/G155 and U159, which adopt the *syn* conformation, are shown in italics. The Mg^2+^ ions are depicted as cyan circles. Non-canonical base-pairs are indicated by red squares. The IS621 residues that interact with the nucleic acids through their main chains are shown in parentheses.

Our structure reveals how the target-binding and donor-binding modules of the bRNA confer DNA specificity to the IS621 ribonucleoprotein complex. The TBL (nucleotides 34–98) comprises stem, LTG, stem loop and RTG regions, which are connected by four linker regions (C47–U49, U59, G72–U73 and U83–U86), whereas the DBL (nucleotides 110–177) similarly comprises stem, LDG, stem loop and RDG regions connected by four linker regions (G121–G122, A131–U132, G155–U156 and U168) (Fig. 2a–c and Supplementary Fig. 2a). The TBL and DBL adopt similar pseudo-symmetric structures with common features, such as flipped-out A nucleotides (A43 and A67 in TBL and A116 and A150 in DBL) and G nucleotides with the *syn* conformation (G48 and G72 in TBL and G121 and G155 in DBL) (Fig. 2a–c and Supplementary Fig. 2b). These A and G nucleotides are highly conserved among the IS110 family elements^2^ and are similarly recognized by the Tnp domains in the four IS621 protomers (Extended Data Fig. 4 and Supplementary Fig. 2c,d). The TBL and DBL also interact with the IS621 recombinase via sugar-phosphate backbone contacts (Extended Data Fig. 4). These structural findings revealed how the IS621 recombinase dimers associate with the structurally distinct TBL and DBL to form two similar ribonucleoprotein complexes.

## DNA recognition mechanism

**Extended Data Fig. 5:**
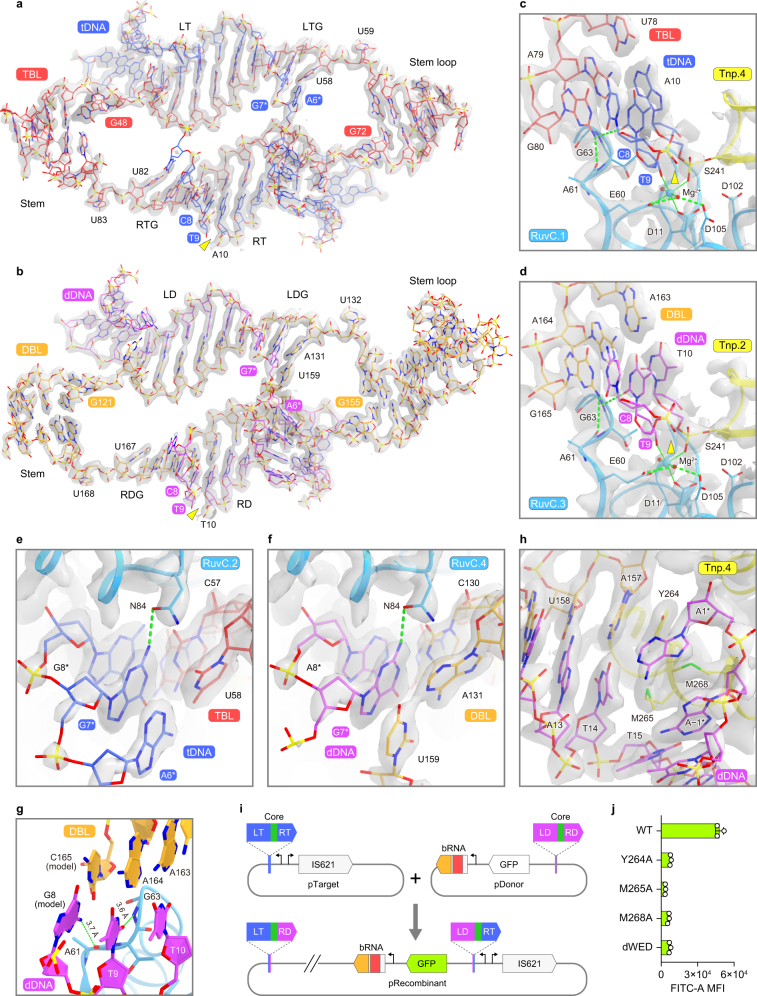
DNA recognition mechanism. (**a**, **b**) Structures of TBL–tDNA (**a**) and DBL–dDNA (**b**). (**c**, **d**) Recognition of the CT core sequences in tDNA (**c**) and dDNA (**d**). (**e**, **f**) Recognition of the CT core-complementary sequences in tDNA (**e**) and dDNA (**f**). (**g**) Modeling of dG8 into the dDNA as part of the core dinucleotide. (**h**) Interaction between the DNA and the hydrophobic wedge. Similar interactions are observed in the four protomers. In (**a**–**f**) and (**h**), cryo-EM density maps are shown as grey semi-transparent surfaces. (**i**) Schematic of the bacterial recombination assays. Successful recombination between pTarget and pDonor places the gene encoding green fluorescent protein (GFP) downstream of the synthetic promoter, resulting in fluorescence. IS621, the IS621 recombinase. (**j**) DNA recombination activities of the WT IS621 and the hydrophobic-wedge mutants in the bacterial recombination assays. Data are shown as mean ± SD for three biological replicates. dWED, Y264A/M265A/M268A.

As predicted by our covariation analysis between bRNA and tDNA–dDNA^2^, LTG (A50–U58) and RTG (G77–G80) in the TBL form base pairs with tT14*–tA6* and tC11–tC8 in the tDNA, respectively, whereas LDG (A123–C130) and RDG (A157–U158/C162–G165) in the DBL form base pairs with dT14*–dG7* and dT14–dA13/dG11–dC8 in the dDNA, respectively (Fig. 2a–c and Extended Data Figs. 4 and 5a,b). We noticed the formation of non-canonical base pairs between TBL G53 and tDNA tT11* and between DBL G161 and dDNA dT12. U159 and U160 are flipped out from the RDG–right donor heteroduplex, with U159 forming a base pair with A131 (Fig. 2a,c). Consistent with our computational and functional analyses^2^, these structural findings demonstrate that the LTG and RTG in the TBL, and the LDG and RDG in the DBL, recognize the bottom and top strands of tDNA and dDNA, respectively, through a unique base-pairing scheme.

The IS621 element and related orthologues contain highly conserved CT core dinucleotides at both ends, and the IS621 recombinase preferentially catalyses recombination between dDNA and tDNA molecules containing the CT core dinucleotide^2^. Whereas the second T nucleotide is almost invariant, the first C nucleotide is less conserved. Indeed, the GT, AT and TT core sequences also supported IS621-mediated recombination in *E. coli* cells^2^. Our structure explains the preference of the IS621 recombinase for the CT core dinucleotides. The tT9 and dT9 nucleobases in the CT cores of tDNA and dDNA are recognized by the main-chain amide group of G63 in the RuvC.1 and RuvC.3 domains, respectively, explaining the stricter conservation of a T nucleotide at position 9 (Extended Data Figs. 4 and 5c,d). Although the tC8 and dC8 nucleobases in the CT core do not contact the protein, the G80 (RTG) and G165 (RDG) nucleobases form hydrogen bonds with the main-chain carbonyl group of A61 in the RuvC.1 and RuvC.3 domains, respectively. Meanwhile, their complementary tG7* and dG7* nucleobases are recognized by the N84 residue in the RuvC.2 and RuvC.4 domains, respectively (Extended Data Figs. 4 and 5e,f). Modelling of G at position 8 in the tDNA and dDNA suggested that the G nucleobase at this position can form hydrogen bonds with the main-chain carbonyl group of A61, whereas A or T at position 8 would be incapable of this interaction (Extended Data Fig. 5g), consistent with the preference of IS621 for C/G over A/T at the first position in the core dinucleotide^2^. Together, these findings explain the core dinucleotide preference of the IS621 recombinase.

Our structure also reveals how the Tnp domains destabilize the DNA duplexes to promote bRNA-mediated recognition of both tDNA and dDNA. Y264, M265 and M268 in the Tnp.1 and 2 and Tnp.3 and 4 domains wedge between the tA1–tT14* and tA14–tT1* pairs in tDNA and between the dA1–dT14* and dT14–dA1* pairs in dDNA, respectively (Extended Data Figs. 4 and 5h). Mutations of these three residues substantially reduced the IS621-mediated recombination in *E. coli* (Extended Data Fig. 5i,j), confirming the functional importance of the hydrophobic wedge. Hydrophobic residues are quite common at these three positions among IS110 orthologues (Supplementary Fig. 1), suggesting that the hydrophobic wedge is a conserved feature of the IS110 recombinases.

## Synaptic complex formation

**Fig. 3:**
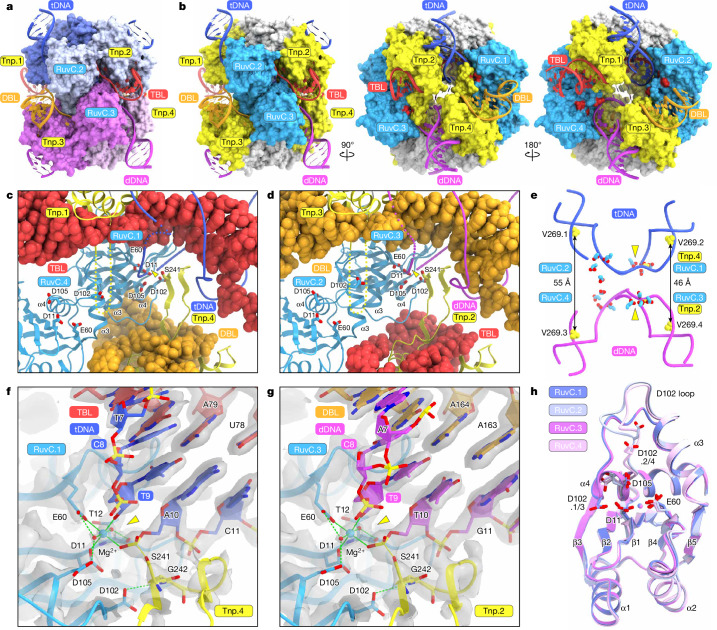
Synaptic complex formation. **a**,**b**, Surface representations of the IS621 synaptic complex, coloured according to the protomers (**a**) and domains (**b**). In **b**, the catalytic residues are coloured in red. **c**,**d**, Active sites formed by RuvC.1 and Tnp.4 (**c**) and RuvC.3 and Tnp.2 (**d**). The TBL and DBL are shown as space-filling models. DNA cleavage sites are indicated by yellow triangles. Disordered regions are indicated by dotted lines. **e**, Locations of the active sites relative to the tDNA and dDNA. **f**,**g**, Close-up views of the active sites formed by RuvC.1 and Tnp.4 (**f**) and RuvC.3 and Tnp.2 (**g**). Cryo-EM density maps are shown as grey semi-transparent surfaces. The Mg^2+^ ions and water molecules are depicted as cyan and red spheres, respectively. Hydrogen and coordinate bonds are shown as green dashed and solid lines, respectively. DNA cleavage sites are indicated by yellow triangles. **h**, Superimposition of the RuvC domains in the four IS621 protomers. The Mg^2+^ ions are depicted as spheres.

**Extended Data Fig. 6:**
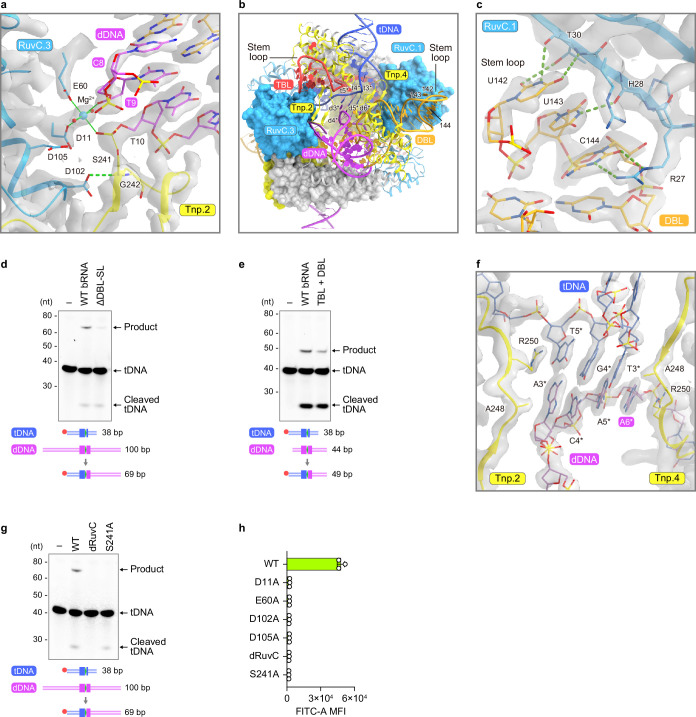
Synaptic complex formation. (**a**) Interactions between RuvC.3 and Tnp.2. Similar interactions are observed between RuvC.1 and Tnp.4. (**b**) Interface between the IS621–TBL–tDNA and IS621–DBL–dDNA dimeric complexes. Nucleotides tT3*–tT5* in the target DNA and nucleotides dA3*–dA6* in the donor DNA are labeled as t3*–t5* and d3*–d6*, respectively. (**c**) Interactions between RuvC.1 and DBL-SL. (**d**) In vitro DNA recombination activities of IS621 in complex with the WT bRNA or the ΔDBL-SL bRNA mutant, in which nucleotides C137–G147 were replaced with GAAA. The tDNA (38 bp) substrate was labeled with Cy5 at the 5′ end of the top strand. The Cy5-tDNA (38 bp) and non-labeled dDNA (100 bp) were incubated with the IS621–bRNA complex at 37 °C for 1 h, and then the reaction was analyzed using an 18% TBE–urea gel. Recombination between the Cy5-tDNA and dDNA yields a 69-bp Cy5-labeled product. Experiments were repeated at least three times with similar results. (**e**) In vitro DNA recombination activities of IS621 in complex with the WT bRNA or separated TBL (nucleotides 31–104) and DBL (nucleotides 99–177). The tDNA (38 bp) substrate was labeled with Cy5 at the 5′ end of the top strand. The Cy5-tDNA (38 bp) and non-labeled dDNA (44 bp) (containing 6-nt mismatches in their top strands) were incubated with the IS621–bRNA complex at 37 °C for 1 h, and then the reaction was analyzed using an 18% TBE–urea gel. Recombination between the Cy5-tDNA and dDNA yields a 49-bp Cy5-labeled product. Experiments were repeated at least three times with similar results. (**f**) Base-pairing between tDNA and dDNA. In (**a**), (**c**), and (**f**), cryo-EM density maps are shown as grey semi-transparent surfaces. (**g**) DNA recombination activities of the WT IS621 and the active-site mutants in the in vitro recombination assays. The tDNA (38 bp) substrate was labeled with Cy5 at the 5′ end of the top strand. The Cy5-tDNA (38 bp) and non-labeled dDNA (100 bp) were incubated with the IS621–bRNA complex at 37 °C for 1 h, and then the reaction was analyzed using an 18% TBE–urea gel. Recombination between the Cy5-tDNA and dDNA yields a 69-bp Cy5-labeled product. Experiments were repeated at least three times with similar results. dRuvC, D11A/E60A/D102A/D105A. (**h**) DNA recombination activities of the WT IS621 and the active-site mutants in the bacterial recombination assays. Data are shown as mean ± SD for three biological replicates.

The IS621 synaptic complex appears to result from the assembly of the two IS621.1/2–TBL–tDNA and IS621.3/4–DBL–dDNA dimeric complexes (Fig. 3a,b). The RuvC domains have a primary role in synaptic complex formation, with the two dimers contacting each other through RuvC–RuvC interactions (Fig. 3c,d and Supplementary Video 2). As described above, the S241 loops in Tnp.4 and Tnp.2 penetrate the DBL and TBL to interact with the D102 loops in RuvC.1 and RuvC.3, respectively, thereby forming the composite active sites comprising D11, E60 and D105 (RuvC) and S241 (Tnp) (Fig. 3c,d and Supplementary Video 2). The D102 residues from RuvC.1 and RuvC.3 form hydrogen bonds with the conserved G242 residues of Tnp.4 and Tnp.2 to stabilize the conformation of the S241 loops (Extended Data Fig. 6a). In contrast to this physical proximity and interaction between RuvC.1 and Tnp.4 and between RuvC.3 and Tnp.2, the other two Tnp domains are farther away from their respective RuvC partners, rendering their S241 loops incapable of forming the same kind of composite active site (Fig. 3c–e).

The synaptic complex is further stabilized by the interaction between the stem loop of the DBL (DBL-SL) and RuvC.1 (Extended Data Fig. 6b). U142, U143 and C144 in DBL-SL form base-specific contacts with T30, H28 and R27 in RuvC.1, respectively (Extended Data Fig. 6c). The replacement of nucleotides 137–147 of DBL-SL with a GAAA tetraloop reduced the in vitro recombination activity of the system (Extended Data Fig. 6d), indicating the functional importance of these interactions for synaptic complex assembly. By contrast, the corresponding stem loop of the TBL (TBL-SL) is shorter than DBL-SL and does not contact RuvC.3 (Extended Data Fig. 6b). Separate TBL and DBL molecules similarly supported IS621-mediated recombination in vitro (Extended Data Fig. 6e), lending further confidence to the notion that the TBL and DBL within a single synaptic complex are derived from two distinct bRNA molecules. We observed that tT3*, tG4* and tT5* of the tDNA form base pairs with dA5*, dC4* and dA3* of the dDNA, respectively (Extended Data Fig. 6b,f). However, donor–target base pairing at these positions did not affect recombination efficiencies in our bacterial recombination assays^2^, indicating that these donor–target base pairs are not functionally important.

Given the structural similarity between the IS621.1/2–TBL–tDNA and IS621.3/4–DBL–dDNA dimeric complexes (Supplementary Fig. 3a,b), we wondered whether IS621 can mediate target–target and/or donor–donor recombination. We found that IS621 is capable of donor–donor, but not target–target, recombination in vitro (Supplementary Fig. 3c). A model of the IS621–TBL–tDNA tetrameric complex suggests that two IS621–TBL–tDNA dimeric complexes cannot form the tetrameric synaptic complex, due to the lack of the DBL-SL–RuvC interactions (Supplementary Fig. 3d), thus explaining why IS621 cannot mediate target–target recombination. By contrast, a model of the IS621–DBL–dDNA tetrameric complex indicates that the DBL-SL–RuvC interaction on both constituent dimers may promote synaptic complex formation (Supplementary Fig. 3e), thereby potentially allowing donor–donor recombination^2^. We observed rare instances of donor–donor recombination, but not target–target genomic rearrangements, in *E. coli*^2^, suggesting that the low target–target recombination efficiency of IS621 biologically contributes to preventing unintended genomic rearrangements. Together, our analyses reveal that the IS621 synaptic complex is stabilized by the protein–protein and protein–nucleic acid interactions between the two distinct dimers.

## Active site architecture

In the RuvC.1–Tnp.4 composite active site, the top strand of tDNA is cleaved between tT9 and tA10, adjacent to the CT core (tC8–tT9) (Fig. 3f). S241 in Tnp.4 forms a covalent 5′-phosphoserine intermediate with tA10, generating a 3′-OH group on tT9. This 3′-OH group and the phosphate group of tA10–S241.4 are coordinated by a Mg^2+^ ion, which is further coordinated by D11 and E60 in RuvC.1 and two water molecules, which in turn form hydrogen bonds with E60, D105 and T12 in RuvC.1. In the RuvC.2–Tnp.3 active site, the top strand of dDNA is cleaved between dT9 and dA10, thereby generating a 3′-OH group in dT9 and a 5′-phosphoserine intermediate between dA10 and S241.2, which are stabilized by a Mg^2+^ ion bound to RuvC.3 (Fig. 3g). Although D11 and E60 assume similar conformations in all four RuvC domains, D102 and D105 adopt distinct conformations in RuvC.1 and RuvC.3 compared with RuvC.2 and RuvC.4, as the RuvC.1 and RuvC.3 domains neither interact with the S241 loop nor participate in composite active site formation (Fig. 3h). The mutation of D11, E60, D102, D105 or S241 abolished IS621-mediated recombination in our in vitro and bacterial recombination assays (Extended Data Fig. 6g,h), confirming the essential role of the RuvC–Tnp composite active site. Together, these findings revealed that D11, E60, D102 and D105 in the RuvC.1 and RuvC.3 domains and S241 in the opposite Tnp.4 and Tnp.2 domains form the composite active sites that cleave the top strands of tDNA and dDNA adjacent to the CT core, respectively, thereby generating the 5′-phosphoserine intermediates during IS621-catalysed recombination.

## Handshake base pairing

The tyrosine recombinase Cre forms a tetrameric synaptic complex with two DNA molecules containing *lox*P sequences and cleaves the top strands of both DNA molecules, forming covalent 3′-phosphotyrosine–DNA linkages and free 5′-OH groups^12^. The 5′-OH groups then attack the 3′-phosphotyrosine intermediates in the opposite DNA molecule to achieve top-strand exchange, creating a Holliday junction intermediate that is resolved by cleavage of the bottom strand followed by a second strand exchange to complete the recombination reaction^12^. Thus, the 5′-phosphoserine–DNA intermediates that we observed in the IS621 structure suggest that the top strands of tDNA and dDNA are exchanged after cleavage. However, the present structure is trapped in the pre-strand exchange state, probably because strand exchange is prevented due to the base pairs between the TBL/DBL and the mismatched nucleotides that we introduced for cryo-EM analysis (A81:tT7, U82:tT6 and G166:dA7).

**Extended Data Fig. 7:**
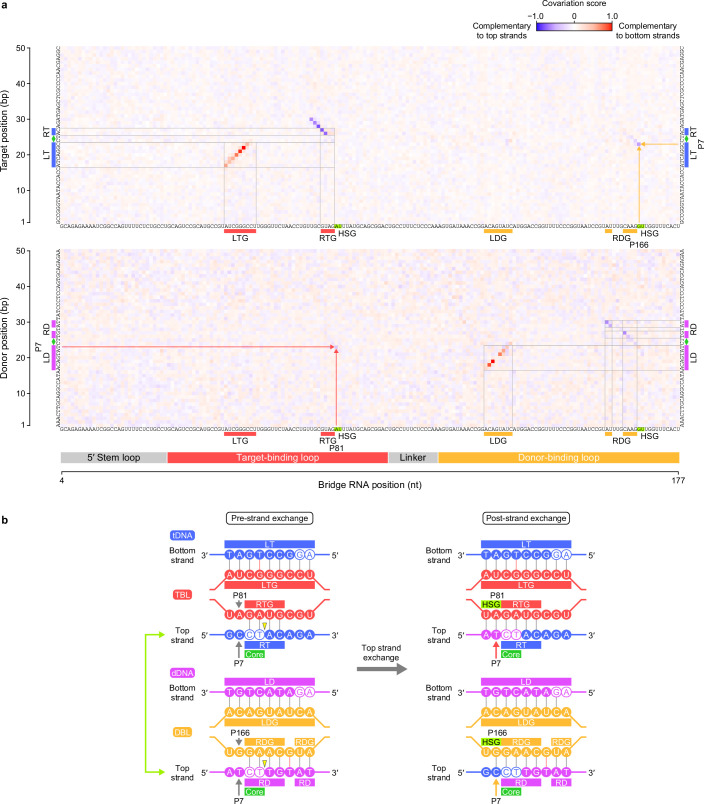
Covariation analysis. (**a**) Nucleotide covariation and base-pairing potential between bRNAs and their target (top) and donor (bottom) sequences, calculated from 5,511 bRNA–target pairs and 2,201 bRNA–donor pairs, as described previously^2^. The IS621 bRNA sequence is shown across the x-axis. Covariation scores calculated from thousands of IS110 orthologues are colored according to strand complementarity, with −1 (blue) representing high covariation and a bias toward top strand base-pairing, 1 (red) representing high covariation and a bias toward bottom strand base-pairing, and 0 indicating no detectable covariation. Regions with substantial covariation signals indicating base-pairing for IS621 are boxed. These data indicated base-pairing potential between TBL-HSG P81 and dDNA P7 (red arrow) and DBL-HSG P166 and tDNA P7 (orange arrow) in IS621, suggesting the functional importance of the base-pairing at these positions across the IS110 orthologues. HSG, handshake guide. (**b**) Schematic of the predicted TBL–tDNA and DBL–dDNA base-pairing patterns before and after the strand exchange.

Our nucleotide covariation analysis^2^ detected uncharacterized covariation signals indicating base-pairing potential between position 7 of the tDNA and dDNA with their non-cognate bRNA loops across hundreds of IS110 orthologues (that is, position 7 in tDNA and position 7 in dDNA may base pair with position 166 in the RDG and position 81 in the RTG, respectively) (Extended Data Fig. 7a,b). These observations suggest that the formation of new base pairs at these positions after strand exchange is important for promoting the strand exchange process. We named these nucleobases (positions 81–82 and 166–167 in the bRNA) handshake guides (HSGs), due to their potential role in helping to ‘introduce’ the donor top strand to the TBL and the target top strand to the DBL for top-strand exchange.

**Extended Data Fig. 8:**
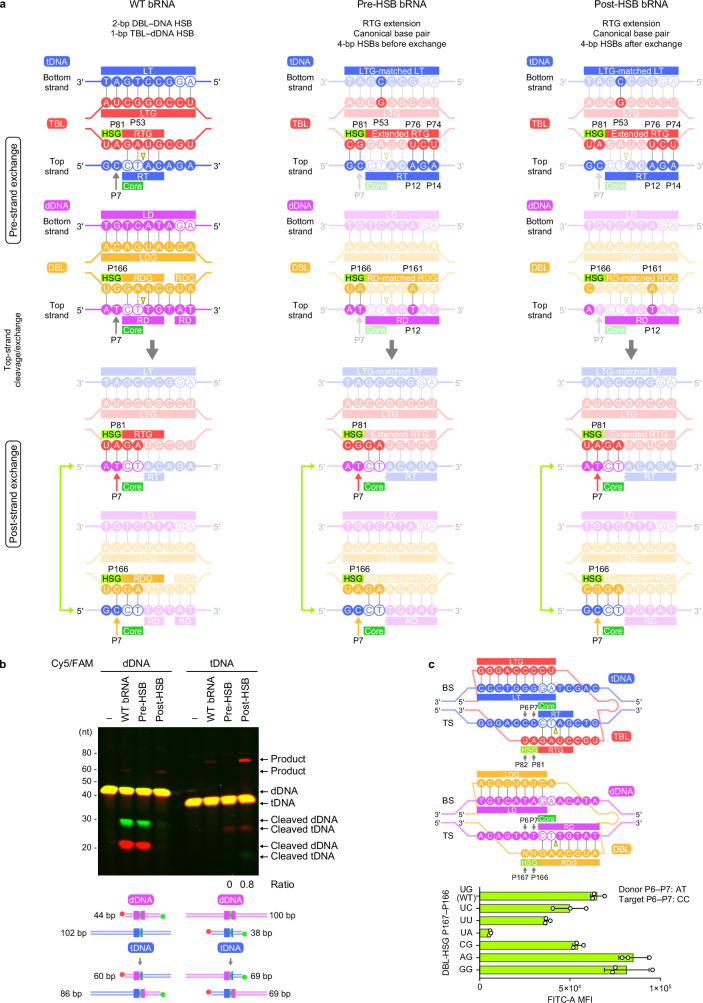
Effects of handshake base-pairing. (**a**) Schematics of the TBL/DBL and tDNA/dDNA sequences used for cryo-EM analysis and in vitro recombination assays. The pre- and post-HSB (handshake base-pairing) bRNAs stabilize the synaptic complex in the pre- and post-strand exchange states, respectively. Mutated nucleotides in the pre- and post-HSB bRNAs and their complementary DNA nucleotides are highlighted. (**b**) Effects of handshake base-pairing on in vitro DNA activity of IS621. Three bRNAs with different HSGs, A81/U82/G166/U167 (WT), G81/C82/A166/U167 (pre-HSB) or A81/U82/G166/C167 (post-HSB), were used for the in vitro DNA recombination experiments. The tDNA (38 bp) and dDNA (44 bp) substrates were labeled with Cy5 and FAM at the 5′ ends of the top and bottom strands, respectively. The Cy5/FAM-tDNA (38 bp) and Cy5/FAM-dDNA (44 bp) were mixed with the non-labeled dDNA (100 bp) and tDNA (102 bp), respectively. The DNA substrates were incubated with the IS621–bRNA complex at 37 °C for 1 h, and the reaction was then analyzed using an 18% TBE–urea gel. Recombination between the Cy5/FAM-dDNA and tDNA and between the Cy5/FAM-tDNA and dDNA yields 60- and 69-bp Cy5-labeled products, respectively. For the results with the labeled tDNA and pre- and post-HSB bRNAs, the band intensities of the product and cleaved DNAs were quantified, and the recombination ratios (product DNA / product DNA + cleaved DNA) were calculated. Experiments were repeated at least three times with similar results. (**c**) Effects of handshake base-pairing between the DBL-HSG and dDNA on IS621-mediated DNA recombination in *E. coli*. Data are shown as mean ± SD for three biological replicates.

To test the effect of handshake base pairing (HSB) on IS621-mediated recombination, we performed in vitro recombination experiments using three bRNAs with different HSG sequences, along with tDNA (with tG6 and tC7) and dDNA (with dA6 and dT7) (Extended Data Fig. 8a). The wild-type (WT) bRNA produced the expected recombination product, but also substantial amounts of cleaved tDNA and dDNA, suggesting that strand exchange was less efficient than top-strand cleavage (Extended Data Fig. 8b). In the second bRNA variant (post-HSB), we extended the RTG–right target complementarity by three additional base pairs (U74:tA14–U76:tA12), which enhanced the recombination efficiency in *E. coli*^2^. In addition, we reprogrammed U167 in the HSG to C, so that the HSGs of the TBL and DBL base pair with positions 6–7 in dDNA and tDNA after strand exchange, respectively (that is, TBL–dDNA and DBL–tDNA non-cognate base pairs). In contrast to the WT bRNA, the post-HSB bRNA strongly supported DNA recombination in vitro and drastically reduced the production of cleaved dDNA (Extended Data Fig. 8b). In the third bRNA variant (pre-HSB), we mutated the HSGs of each binding loop to base pair with their cognate DNA before strand exchange (that is, TBL–tDNA and DBL–dDNA cognate base pairs). The pre-HSB bRNA mediated robust cleavage of the top strands of tDNA and dDNA, but did not lead to productive in vitro recombination (Extended Data Fig. 8b). These results indicate that the base pairing between the HSGs of the TBL and DBL and their non-cognate DNA facilitates strand exchange, whereas the base pairing between the HSGs and their cognate DNA inhibits strand exchange.

Next, we explored whether the potential of the DBL HSG to base pair with tDNA would affect the efficiency of recombination in *E. coli*. Mirroring our results in vitro, we observed that the G166A mutation in the DBL, which enables base pairing to dT7 of the dDNA, dramatically reduced the recombination efficiency, whereas G166 (WT), G166C and G166U allowed functional recombination (Extended Data Fig. 8c). Furthermore, reprogramming U167 to G, which can base pair with tC6 of the tDNA after strand exchange, enhanced the recombination efficiency (approximately 1.2-fold). Together, our in vitro and in cellulo recombination assays revealed that the HSG positions can be programmed as a key determinant of recombination efficiency.

To understand how the pre-HSB bRNA precludes recombination, we determined the cryo-EM structure of the synaptic complex with the pre-HSB bRNA (Supplementary Fig. 4a–d). The structure is similar to our original structure, with the top strands of tDNA and dDNA cleaved to form 5′-phosphoserine intermediates (Supplementary Fig. 4e–h). However, in contrast to the original structure, the HSGs in the TBL and DBL form the expected base pairs with the tDNA and dDNA, respectively, thereby impeding top-strand exchange (Supplementary Fig. 4e–h). Thus, our biochemical and structural observations indicate that the cognate base pairing with the HSGs traps the IS621 synaptic complex in the pre-strand exchange ‘locked’ state and does not allow recombination to proceed. Collectively, these findings strongly support our hypothesis that HSGs, which are conserved in many natural IS110 systems, enable the top strands of both dDNA and tDNA to form base pairs with the opposite bRNA loop, thereby facilitating strand exchange.

## Strand exchange mechanism

**Fig. 4:**
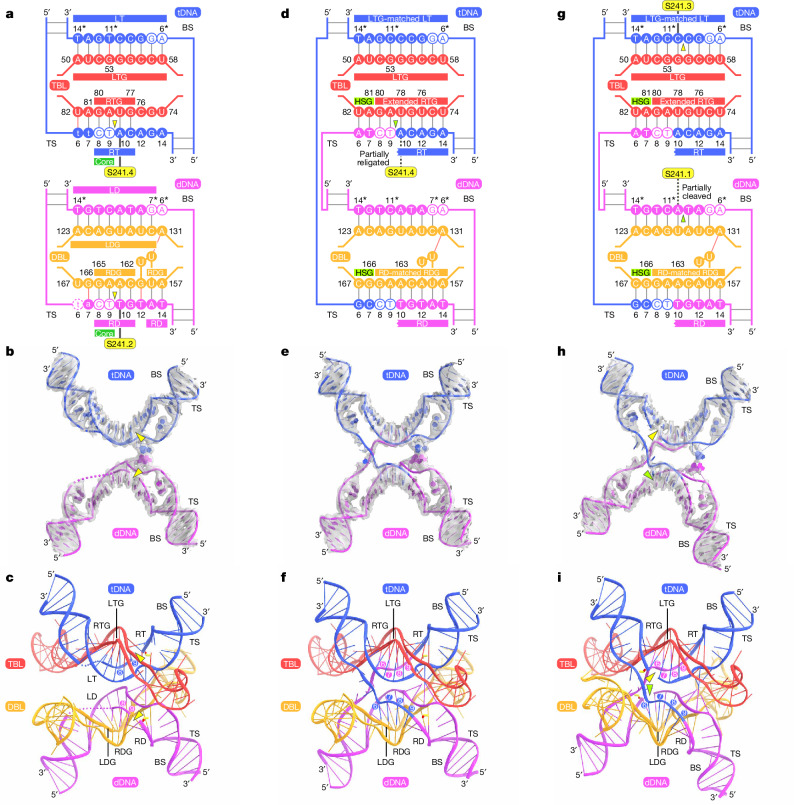
Strand exchange mechanism. **a**–**i**, Schematics of TBL–tDNA and DBL–dDNA and the structures of tDNA and dDNA, and TBL–tDNA and DBL–dDNA, in the IS621 synaptic complexes in the pre-strand exchange state (the complex with the WT bRNA for comparison) (**a**–**c**); the post-strand exchange, Holliday junction intermediate state (state 1) (**d**–**f**); and the post-strand exchange, Holliday junction resolution state (state 2) (**g**–**i**). DNA cleavage sites are indicated by yellow triangles, whereas partial cleavage and religation are indicated by green triangles. In **b**,**e**,**h**, cryo-EM density maps are shown as grey semi-transparent surfaces. In **b**,**c**, disordered regions are indicated by dotted lines. In **c**,**f**,**i**, positions 6–9 in the dDNA and tDNA are numbered.

**Extended Data Fig. 9:**
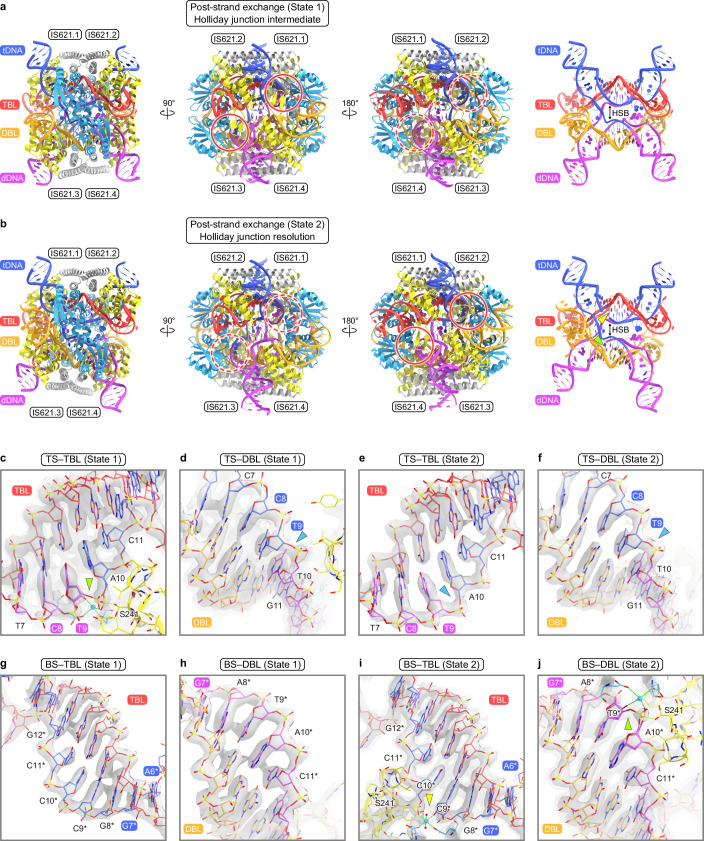
Structures of the IS621 synaptic complexes in the post-strand exchange states. (**a**, **b**) Structures of the IS621 synaptic complexes in the post-strand exchange states (state 1) (**a**) and (state 2) (**b**). The active sites with the ordered and disordered S241 residues are indicated by red solid and dashed circles, respectively. (**c**–**j**) Close-up views of TS–TBL (state 1) (**c**), TS–DBL (state 1) (**d**), TS–TBL (state 2) (**e**), TS–DBL (state 2) (**f**), BS–TBL (state 1) (**g**), BS–DBL (state 1) (**h**), BS–TBL (state 2) (**i**), and BS–DBL (state 2) (**j**). Cryo-EM density maps are shown as grey semi-transparent surfaces. The Mg^2+^ ions are depicted as cyan spheres. In (**c**), (**d**), (**i**), and (**j**), the density maps are contoured at two different levels. The top strand is partially religated at the RuvC.1–Tnp.4 active site in state 1 (green arrow) (**c**), whereas the top strand is fully religated at the RuvC.1–Tnp.4 active site in state 2 (cyan arrow) (**e**). The top strands are fully religated at the RuvC.3–Tnp.2 active site in states 1 and 2 (cyan arrows) (**d**, **f**). The bottom strands of tDNA and dDNA are not cleaved in state 1 (**g**, **h**). The bottom strand of tDNA is fully cleaved at the RuvC.2–Tnp.3 active site in state 2 (yellow arrow) (**i**), whereas that of dDNA is partially cleaved at the RuvC.4–Tnp.1 active site in state 2 (green arrow) (**j**). TS, top strand; BS, bottom strand.

To explore the next steps of the IS621-catalysed recombination mechanism, we determined the cryo-EM structure of the IS621 synaptic complex containing the post-HSB bRNA with the following features that would facilitate recombination: (1) RTG extension (positions 74–76), in which U74, C75 and U76 in the TBL base pair with tA14, tG13 and tA12 in the tDNA, respectively to stabilize the synaptic complex; (2) replacement of non-canonical base pairs observed in the original structure (positions 53 and 161); and (3) one additional handshake base in the DBL (position 167) (Fig. 4a–i and Supplementary Fig. 5a–g). Our cryo-EM analysis revealed two distinct conformational states: state 1 (2.9 Å resolution) and state 2 (2.7 Å resolution) (Fig. 4d–i, Extended Data Fig. 9a,b and Supplementary Video 3). As expected, in both states 1 and 2, U74–U76 of bRNA base pair with tA14–tA12 of tDNA, whereas G53 and A161 of bRNA form canonical base pairs with tC11* of tDNA and dT12 of dDNA, respectively (Fig. 4d,g). Of note, the top strands of tDNA and dDNA are cleaved downstream of the CT core (between tT9 and tA10 in tDNA and between dT9 and dT10 in dDNA) and then exchanged, resulting in the formation of new TBL–dDNA and DBL–tDNA base pairs, as predicted by our HSG experiments (Fig. 4d–i). The RDG remains bound to positions 10–14 of the dDNA, while forming new base pairs with the incoming core (tC8–tT9) of the tDNA top strand. Adjacent to this interaction, positions 166–167 of the DBL (the HSG region) also base pair with positions 6–7 of the tDNA. Similarly, the RTG remains bound to positions 10–14 of the tDNA, while forming new base pairs with the incoming core (dC8–dT9) of the dDNA top strand. In addition, positions 81–82 of the TBL (the other HSG region) base pair with positions 6–7 of the dDNA.

In both structures, phosphodiester bonds are formed between dDNA dT9 and tDNA tA10 and between tDNA tT9 and dDNA dT10, with the S241.4 and S241.2 residues dissociated from tDNA tA10 and dDNA dT10, respectively, creating a Holliday junction intermediate (Extended Data Fig. 9c–f). In state 1, the phosphodiester bond between dDNA dT9 and tDNA tA10 exhibits relatively weak density, with S241.4 located close to tDNA tA10 (Extended Data Fig. 9c), suggesting partial religation between dDNA dT9 and tDNA tA10. Together, these observations support the notion that IS621 cleaves the top strands of tDNA and dDNA, followed by strand exchange and religation to form a Holliday junction intermediate.

In state 1, the S241 loops in Tnp.1 and 3 are disordered and the bottom strands of tDNA and dDNA are intact, as in the pre-strand exchange structure (Fig. 4d–f and Extended Data Fig. 9a,g,h). By contrast, in state 2, the S241 loops in Tnp.1 and 3 become ordered and the bottom strands of tDNA and dDNA are cleaved between tC9* and tC10* and between dT9* and dA10* at the RuvC.2–Tnp.3 and RuvC.4–Tnp.1 active sites, with the S241.3 and S241.1 residues covalently linked to tDNA tC10* and dDNA dA10*, respectively (the bottom strand of dDNA is partially cleaved) (Fig. 4g–i and Extended Data Fig. 9b,i,j). Accordingly, the Holliday junction intermediate is resolved by the cleavage of the tDNA and dDNA bottom strands at the RuvC–Tnp composite active sites of the two IS621 protomers that did not participate in top-strand cleavage (RuvC.2–Tnp.3 and RuvC.4–Tnp.1). We conclude that the IS621 synaptic complex structures in states 1 and 2 represent the post-strand exchange, the Holliday junction intermediate state and the Holliday junction resolution state, respectively. Together, our structural and functional data reveal a step-by-step mechanism explaining how the IS621 recombinase and its bispecific bRNA orchestrate programmable recombination between donor and target DNA.

## Discussion

**Fig. 5:**
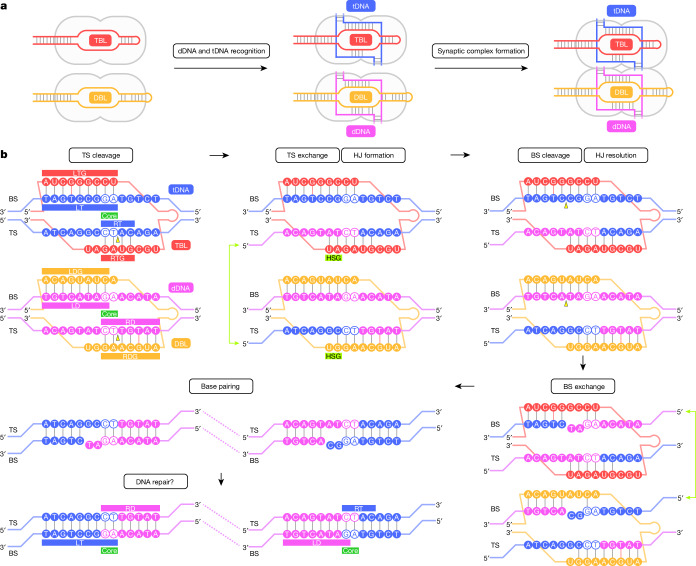
Bridge recombination mechanism. **a**,**b**, Proposed mechanisms of the IS621 synaptic complex formation (**a**) and the IS621-mediated DNA recombination (**b**). Two IS621 recombinase molecules bind to the TBL and DBL from two different bRNA molecules to form the IS621–TBL and IS621–DBL dimeric complexes, respectively. IS621–TBL and IS621–DBL recognize tDNA and dDNA, respectively, and then IS621–TBL–tDNA and IS621–DBL–dDNA form the tetrameric synaptic complex. In the synaptic complex, the top strands of tDNA and dDNA are cleaved at the RuvC–Tnp active sites, with the catalytic S241 residues forming covalent 5′-phosphoserine intermediates. The top strands are then exchanged and religated to form a Holliday junction (HJ) intermediate, which is resolved by the cleavage of the bottom strands at the RuvC–Tnp active sites. It is possible that the bottom strands are exchanged, and mismatched nucleotides are excised and repaired in *E. coli* cells, thereby completing the recombination. DNA cleavage sites are indicated by yellow triangles. Canonical and non-canonical base pairs are indicated by grey and red lines, respectively.

The IS621 recombinase of the IS110 family utilizes a bispecific bRNA with independently programmable target-binding and donor-binding loops to orchestrate recombination between diverse pairs of DNA sequences^2^. Here we provide the first view of this new class of RNA-guided DNA recombinase complexes, illuminating how the IS621 recombinase brings together two bRNA-specified DNA molecules to catalyse their recombination (Fig. 5a,b).

**Extended Data Fig. 10:**
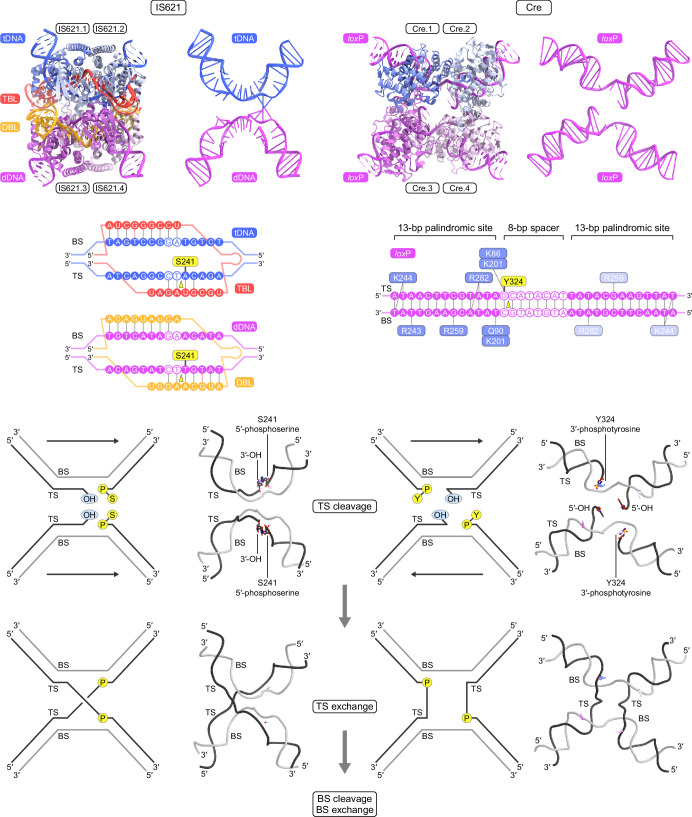
Comparison of IS621 and Cre. Comparison of the synaptic complex structures, DNA recognition mechanisms, and DNA recombination mechanisms between IS621 and Cre (PDB: 1CRX and 3CRX). In the recombination reactions catalysed by IS621 and Cre, the top strands in two DNA molecules are cleaved, forming covalent protein–DNA intermediates. DNA cleavage sites are indicated by yellow triangles. Note that the relative angles between the two DNA molecules differ by ~180° between the synaptic complexes of IS621 and Cre, resulting in the opposite orientations of their HJ intermediates (parallel for IS621 and antiparallel for Cre). The orientations of the two DNA molecules are indicated by arrows. TS, top strand; BS, bottom strand.

Like site-specific recombinases such as Cre^12^, the IS621 recombinase catalyses the recombination reaction consisting of top-strand cleavage and exchange, Holliday junction formation, and bottom-strand cleavage and exchange (Extended Data Fig. 10 and Supplementary Discussion). However, IS621 uses a distinct domain architecture, RuvC–Tnp composite active sites and a bRNA guide, thereby distinguishing it from all known recombinase systems^3,11,12,18,19^ (Supplementary Fig. 6). Although many recent studies have expanded the known universe of RNA-guided enzymes^20–33^, our discovery of the IS110 bridge RNA illustrates a conceptually distinct way by which enzymes utilize RNA-mediated DNA recognition to manipulate nucleic acids (Supplementary Discussion). Not only does IS621 represent the first example of a single-protein RNA-guided DNA recombinase but its bRNA also contains two distinct guide segments that base pair with both the top and bottom strands of the dDNA and tDNA — a unique feature among all characterized RNA-guided systems to date.

The cryo-EM structures that we determined in this study represent the insertion step in the IS621 transposition cycle, where the circular intermediate (dDNA) recombines into the genomic target site (tDNA). Other stages of the IS621 transposition cycle remain to be mechanistically investigated. Notably, the IS621 system comprises only a single, small protein (326 amino acids) and a single non-coding RNA molecule (177 nt) to accomplish modular and programmable recognition of dDNA and tDNA and their recombination — without introducing double-stranded DNA breaks. Collectively, this work provides fundamental insights into transposable element spreading and RNA-guided enzymatic mechanisms, and offers a mechanistic framework to understand and engineer bridge recombination systems as flexible tools for genome design.

## Methods

### Protein and RNA preparation

The IS621 recombinase gene was cloned into a modified pFastBac1 expression vector (Thermo Fisher Scientific), which encodes an N-terminal His_8_ tag, a Twin-Strep tag and a human rhinovirus 3C protease cleavage site (Supplementary Table 1). The IS621 protein was expressed in Sf9 cells (Thermo Fisher Scientific), using the Bac-to-Bac baculovirus expression system (Thermo Fisher Scientific). Sf9 cells were cultured in Sf900II medium (Thermo Fisher Scientific), infected with the recombinant baculovirus at a density of approximately 2 × 10^6^ cells per millilitre, and then incubated at 27 °C for 48 h. The cells were collected by centrifugation at 5,000*g* and stored at −80 °C before use. The Sf9 cells were lysed by sonication in lysis buffer (20 mM Tris-HCl (pH 7.5), 1 M NaCl, 2 mM MgCl_2_, 2% Triton X-100 and protease inhibitor cocktail), and the lysate was clarified by centrifugation at 40,000*g*. The supernatant was mixed with Strep-Tactin XT resin (IBA) at 4 °C for 1 h. The resin was washed with wash buffer (20 mM Tris-HCl (pH 7.5), 500 mM NaCl, 2 mM MgCl_2_, 3 mM 2-mercaptoethanol and 10% glycerol), and the protein was eluted with wash buffer containing 80 mM biotin. The eluted protein was purified by size-exclusion chromatography on a Superdex 200 Increase 10/300 GL column (Cytiva), equilibrated with buffer (20 mM Tris-HCl (pH 7.5), 500 mM NaCl, 1 mM dithiothreitol (DTT), 10% glycerol and 2 mM MgCl_2_). The peak fractions were collected and stored at −80 °C until use. The S241A and D11A/E60A/D102A/D105A mutants were similarly expressed and purified. The bRNAs were transcribed in vitro with T7 RNA polymerase and purified by 10% denaturing (7 M urea) PAGE (Supplementary Table 1).

### Synaptic complex preparation

The IS621–bRNA–dDNA–tDNA synaptic complex was reconstituted by mixing the purified IS621 recombinase, a 177-nt bRNA (177 nt plus 5′-GGG for in vitro transcription), a 44-bp dDNA and a 38-bp tDNA, at a molar ratio of 4:1:1:1. To obtain a synaptic complex with the WT bRNA (pre-strand exchange state), six mismatches were introduced into the top strands of the tDNA and dDNA (Supplementary Table 1). To obtain a synaptic complex with the pre-HSB bRNA (pre-strand exchange locked state) or the post-HSB bRNA (post-strand exchange state), four mismatches were introduced into the top strands of the tDNA and dDNA (Supplementary Table 1). The IS621–bRNA–dDNA–tDNA synaptic complex was purified by size-exclusion chromatography on a Superose 6 Increase 10/300 column (Cytiva), equilibrated with buffer (20 mM Tris-HCl (pH 7.5), 300 mM NaCl, 5 mM MgCl_2_ and 1 mM DTT). The peak fraction containing the synaptic complex was concentrated to 0.5–1 mg ml^−1^, using an Amicon Ultra-4 Centrifugal Filter Unit (MWCO 50 kDa; Millipore). Protein concentrations were measured by the Pierce 660 nm Protein Assay Reagent (Thermo Fisher Scientific).

### Cryo-EM analysis

The grids were glow-discharged in low-pressure air at a 10-mA current in a PIB-10 ion generator (Vacuum Device). The synaptic complex solution was applied to a freshly glow-discharged Quantifoil Holey Carbon Grid (R1.2/1.3, Au, 300 mesh) (SPT Labtech) using a Vitrobot Mark IV system (Thermo Fisher Scientific) at 4 °C, with a waiting time of 10 s and a blotting time of 6 s under 100% humidity conditions. The grids were plunge-frozen in liquid ethane cooled at liquid nitrogen temperature.

The grids containing the synaptic complex with the WT bRNA or the pre-HSB bRNA were transferred to a Titan Krios G3i electron microscope (Thermo Fisher Scientific) running at 300 kV and equipped with a Gatan Quantum-LS Energy Filter (GIF) and a Gatan K3 Summit direct electron detector. The grid containing the synaptic complex with the post-HSB bRNA was transferred to a Titan Krios G4 electron microscope (Thermo Fisher Scientific) running at 300 kV and equipped with a Gatan Quantum-LS Energy Filter and a Gatan K3 Summit direct electron detector. Imaging was performed at a nominal magnification of ×105,000, corresponding to a calibrated pixel size of 0.83 Å per pixel. For the synaptic complex with the WT bRNA, each movie was dose fractionated to 50 frames and recorded using the correlated double-sampling mode at a dose rate of 7.7 e^−^ px^−1^ s^−1^, resulting in a total accumulated exposure of 50 e^−^ Å^−^^2^ of the specimen. For the synaptic complex with the pre-HSB bRNA, each movie was dose fractionated to 48 frames and recorded using the normal mode at a dose rate of 14.7 e^−^ px^−1^ s^−1^, resulting in a total accumulated exposure of 49 e^−^ Å^−^^2^ of the specimen. For the synaptic complex with the post-HSB bRNA, each movie was dose fractionated to 64 frames and recorded using the correlated double-sampling mode at a dose rate of 9.5 e^−^ px^−1^ s^−1^, resulting in a total accumulated exposure of 62 e^−^ Å^−^^2^ of the specimen. The data were automatically acquired using the image-shift method in the EPU software (Thermo Fisher Scientific), with a defocus range of −0.8 to −2.0 μm.

The data were processed using the cryoSPARC v4.3.0 software package^34^. The dose-fractionated movies were aligned using Patch motion correction, and the contrast transfer function (CTF) parameters were estimated using patch-based CTF estimation. For the synaptic complex with the WT bRNA, particles were automatically picked using Blob picker and template picker, followed by reference-free 2D classification to curate particle sets. The particles were further curated by heterogeneous refinement, using the map derived from cryoSPARC ab initio reconstruction as the template. The best-class particle set was refined using non-uniform refinement, yielding a map at 2.58 Å resolution. Local motion correction followed by non-uniform refinement with CTF value optimization yielded a map at 2.52 Å resolution, according to the Fourier shell correlation (FSC) = 0.143 criterion^35^. The local resolution was estimated by BlocRes in cryoSPARC.

For the synaptic complex with the pre-HSB bRNA, particles were automatically picked using template picker, followed by reference-free 2D classification of the WT bRNA sets. The particles were further curated by heterogeneous refinement, using the WT bRNA maps as a template. The best-class particle set was refined using homogeneous refinement and non-uniform refinement, yielding a map at 2.79 Å resolution. Local motion correction followed by non-uniform refinement with CTF value optimization yielded a map at 2.72 Å resolution, according to the FSC = 0.143 criterion. The local resolution was estimated by BlocRes in cryoSPARC.

For the synaptic complex with the post-HSB bRNA, particles were automatically picked using template picker, followed by reference-free 2D classification of the WT bRNA datasets. The particles were further curated by heterogeneous refinement, using the WT bRNA maps as a template. To further distinguish the conformational heterogeneity, the selected particles after homogeneous refinement were divided into four classes using 3D classification. The particle sets in the two selected classes were further refined using homogeneous refinement and heterogeneous refinement. Non-uniform refinement with CTF value optimization yielded maps at 2.88 Å resolution (state 1) and 2.73 Å resolution (state 2), according to the FSC = 0.143 criterion. The local resolution was estimated by BlocRes in cryoSPARC.

### Model building and validation

The models of the IS621–bRNA–dDNA–tDNA synaptic complexes were manually built using COOT^36^, starting from a model predicted by ColabFold^37^. The models were refined using phenix.real_space_refine^38^ and Servalcat^39^ against unsharpened half maps. The models were validated using MolProbity^40^. The statistics of the 3D reconstruction and model refinement are summarized in Extended Data Table 1. The molecular graphics and cryo-EM density map figures were prepared with CueMol (http://www.cuemol.org) or UCSF ChimeraX^41^.

### In vitro recombination assays

For in vitro recombination assays, linear tDNA and dDNA substrates were synthesized by Eurofins Genomics, and labelled with FAM or Cy5 at the 5′ end of the top or bottom strand (Supplementary Table 1). The tDNA (0.1 μM) and dDNA (0.1 μM) substrates were mixed with the pre-incubated IS621–bRNA complex (1.4 μM) in 100 µl buffer (20 mM Tris-HCl (pH 7.5), 300 mM NaCl, 5 mM MgCl_2_ and 1 mM DTT), and then the reactions were incubated at 37 °C for 1 h. The reaction mixture was mixed with proteinase K (Nacalai Tesque) and then boiled at 95 °C for 3 min in denaturing buffer (7 M urea). The samples were analysed by 18% urea-PAGE, and fluorescent signals were imaged using FUSION Solo S (Vilber Bio Imaging). For gel source data, see Supplementary Fig. 7.

### Microscale thermophoresis

Microscale thermophoresis experiments were performed using a Monolith NT.115pico Series instrument (NanoTemper), as previously described^2^. The IS621 recombinase was labelled using the RED-MALEIMIDE 2nd generation cysteine reactive kit (NanoTemper). The labelled protein was eluted in buffer (20 mM Tris-HCl (pH 7.5), 500 mM NaCl, 5 mM MgCl_2_, 1 mM DTT and 0.01% Tween 20). To determine the affinity of the IS621 recombinase for RNA, 20 nM recombinase was incubated with a dilution series (0.076–250 nM) of the bRNA (177 nt), its reverse complement (177 nt) or the bRNA mutant lacking the 5′ stem loop (146 nt). Microscale thermophoresis experiments were performed at 37 °C using premium capillaries (NanoTemper) at medium microscale thermophoresis power with the LED excitation power set to automatic (excitation ranged from 10% to 50%). Data were analysed using the NanoTemper MO.affinity analysis software package, and raw data were plotted using GraphPad Prism 9 (GraphPad).

### Bacterial recombination assays

Bacterial recombination assays were performed, as previously described^2^. In brief, *E. coli* BL21(DE3) cells (NEB) were co-transformed with a pTarget plasmid encoding a target sequence and a T7-inducible IS621 recombinase, and a pDonor plasmid encoding a bRNA, a donor sequence and GFP, such that, upon recombination into pRecombinant, GFP expression would be activated by the synthetic Bba_R0040 promoter adjacent to the target site (Supplementary Table 1). pDonor encodes the WT IS621 donor sequences and pTarget encodes a DNA sequence not found in the *E. coli* genome, and the bRNA was programmed to recombine these two DNA sequences. Co-transformed cells were plated on fresh LB agar containing kanamycin, chloramphenicol and 0.07 mM IPTG to induce recombinase expression. Plates were incubated at 37 °C for 16 h and then at room temperature for 8 h. Hundreds of colonies were scraped from the plate, resuspended in terrific broth and diluted to an appropriate concentration for flow cytometry. About 5 × 10^4^ cells were analysed on a NovoCyte Quanteon Flow Cytometer to assess the fluorescence intensity of GFP-expressing cells (Supplementary Fig. 8). The mean fluorescence intensity of the population (including both GFP^+^ and GFP^−^ cells) was plotted.

### Covariation analysis

Covariation analysis to identify base-pairing potential between bRNA and tDNA or dDNA was performed, as previously described^2^. In brief, IS621 orthologue sequences were searched (blastp) against a curated database of IS110 elements extracted from publicly available genomic sequence archives^42^. Next, a covariance model (CM) of the bRNA primary and secondary structures was used to identify homologues of the bRNA sequence in the non-coding ends of these orthologous sequences^43^. Target and donor sequences centred around the predicted core were extracted. Predicted bRNA sequences were aligned using the cmalign tool in the Infernal package. Two paired alignments were then generated that contained concatenated target and bRNA sequences, and concatenated donor and bRNA sequences. These alignments were analysed using CCMpred (‘-n 100’) to identify covarying nucleotides between target–donor and bRNA sequences^44^. These covariation scores were normalized and multiplied by the sign of a base-pairing concordance score to produce the covariation score scale, which ranged from −1 (top strand base pairing) to +1 (bottom strand base pairing).

### Reporting summary

Further information on research design is available in the Nature Portfolio Reporting Summary linked to this article.

## Online content

Any methods, additional references, Nature Portfolio reporting summaries, source data, extended data, supplementary information, acknowledgements, peer review information; details of author contributions and competing interests; and statements of data and code availability are available at 10.1038/s41586-024-07570-2.

### Supplementary information

Supplementary Information This file contains the Supplementary discussion and Supplementary Figs 1–8.

Reporting Summary

Supplementary Table 1 Nucleic-acids used in the study.

Supplementary Video 1 Structure of the IS621 synaptic complex in the pre-strand exchange state.

Supplementary Video 2 Structures of the RuvC/Tnp composite active sites.

Supplementary Video 3 Structures of the IS621 synaptic complexes in the pre- and post-strand exchange states.

## Figures and Tables

**Extended Data Table 1:**
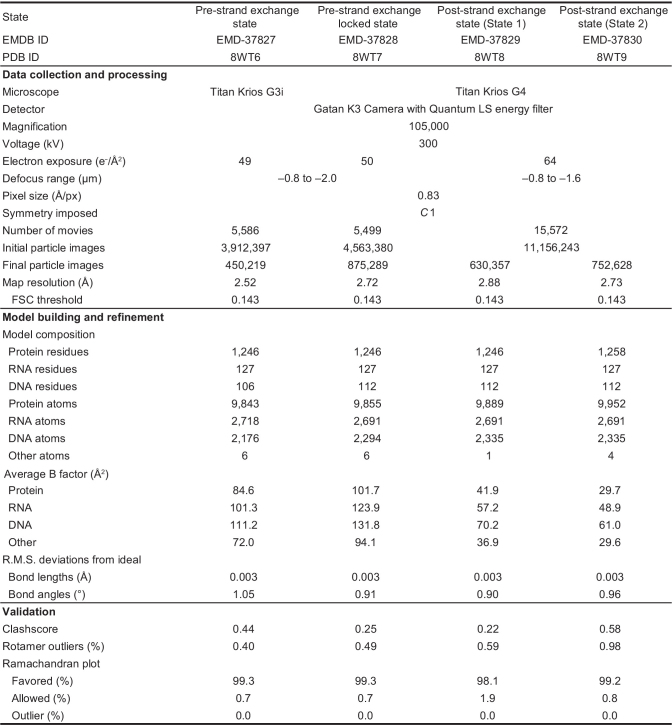
Cryo-EM data collection, refinement, and validation statistics

## Data Availability

Cryo-EM density maps have been deposited in the Electron Microscopy Data Bank under the accession codes EMD-37827 (pre-strand exchange state), EMD-37828 (pre-strand exchange locked state), EMD-37829 (Holliday junction intermediate state) and EMD-37830 (Holliday junction resolution state). Atomic coordinates have been deposited in the Protein Data Bank under IDs 8WT6 (pre-strand exchange state), 8WT7 (pre-strand exchange locked state), 8WT8 (Holliday junction intermediate state) and 8WT9 (Holliday junction resolution state). The raw images have been deposited in the Electron Microscopy Public Image Archive, under the accession code EMPIAR-11804.
