# Supplementary material for: Structural mechanism of bridge RNA-guided recombination
Source: Nature. 2024 Jun 26;630(8018):994–1002. doi: 10.1038/s41586-024-07570-2 (PMC11208158; doi:10.1038/s41586-024-07570-2)
Supplement: Supplementary file 1 — This file contains the Supplementary discussion and Supplementary Figs 1–8. [file 41586_2024_7570_MOESM1_ESM.pdf]

nature portfolio

https://doi.org/10.1038/s41586-024-07570-2

Supplementary information

Structural mechanism of bridge RNA-guided recombination

In the format provided by the authors and unedited

Supplementary Discussion

The structural mechanism we elucidated in this study corresponds to the insertion step of the IS621 life cycle, where the circular dsDNA intermediate recombines into its genomic target site. Intriguingly, while circular intermediates of other IS110 family elements have been detected in their natural systems[5], direct evidence for the IS621 circular form in *E. coli* is still lacking. However, the bRNA is expressed from the predicted circular form of the IS621 element[2], supporting its existence and functional relevance to the transposon life cycle. Importantly, it remains elusive whether the excision process is also mediated by the IS621–bRNA complex and, if so, how it mediates both excision and insertion reactions.

Most transposases use specific protein–DNA contacts to recognize specific inverted repeat sequences in the donor DNA (the transposable element itself) and short motifs in its genomic target site. Following a similar theme, site-specific recombinases, such as the Bxb1 serine recombinase and Cre tyrosine recombinase, employ protein–DNA interactions to recognize specific sequences in two DNA molecules (*att*P/*att*B for Bxb1 and *lox*P for Cre) and catalyse their recombination[3,11,12] (Extended Data Fig. 10 and Supplementary Fig. 6). In stark contrast to all known transposases and recombinases, the IS621 recombinase recognizes both donor and target DNA sequences in an RNA-dependent manner, allowing for broad programmability of these molecules (Extended Data Fig. 10 and Supplementary Fig. 6). Like conventional recombinases such as Bxb1 and Cre, IS621 forms a tetrameric synaptic complex, but possesses the unique RuvC/Tnp composite active sites that have not been observed in other known enzymes. Nonetheless, a structural comparison between IS621 and Cre revealed unexpected similarity between their recombination mechanisms, despite their completely distinct domain architectures (Extended Data Fig. 10). IS621 and Cre both cleave the top strands of two DNA molecules to form covalent phosphoprotein–DNA intermediates, followed by strand exchange, re-ligation, HJ formation, and HJ resolution. However, Cre uses catalytic tyrosine residues for DNA cleavage to generate 3′-phosphotyrosine intermediates and free 5′-OH groups[12], whereas IS621 forms 5′-phosphoserine intermediates to produce free 3′-OH groups. In addition, the relative angles between the two DNA molecules differ by ~180° between the synaptic complexes of IS621 and Cre, resulting in the opposite orientations of their HJ intermediates (parallel for IS621 and antiparallel for Cre). Meanwhile, serine recombinases like Bxb1 rely on a catalytic serine residue but share no structural or mechanistic similarities with IS621, since they cleave all four strands of donor and target DNA to form covalent 5′-phosphoserine–DNA

intermediates within the synaptic complex, and then exchange both strands via rotation of half of the Bxb1–DNA synaptic complex[11] (Supplementary Fig. 6). The IS621 recombinase is therefore distinct from other known enzymes in terms of both DNA recognition and catalytic mechanism.

Our cryo-EM structures also revealed remarkable functional differences between the RuvC domain of IS621 and the DDE domains of typical DDE transposases encoded by most IS elements, such as the IS21 family transposase IstA[18] – although they share an RNase H-like fold[19] (Supplementary Fig. 6). In the RuvC domain of IS621, D11, E60, and D105 in the DEDD motif bind a Mg^{2+} ion and contribute to stabilizing the 5′-phosphoserine intermediate, while D102 interacts with the catalytic serine loop in the Tnp domain in the opposite protomer. In contrast, in the DDE domain of IstA, D124, D187, and E233 in the DDE motif bind two Mg^{2+} ions and contribute to activating a water molecule that nucleophilically attacks the donor DNA, forming free 3′-OH groups in the donor DNA that in turn attack the target DNA. These functional differences explain why the DDE catalytic motifs are widely used by diverse RNase H-like nucleases, while the DEDD motif is specific to the IS110 family recombinases.

The IS621 complex structures presented here provide a framework for future engineering of the bridge recombinase system to improve its efficiency and specificity for genome engineering applications, as extensively explored for CRISPR-Cas nucleases[20]. Indeed, we have already demonstrated that the extension of base-pairing from 4 to 7 bp between the bRNA RTG and the tDNA RT improves the recombination efficiency and specificity in *E. coli*[2]. Our structural and functional observations suggest that DNA invasion is one of the rate-limiting steps in IS621-catalysed recombination. Thus, an important focus of protein engineering efforts may be to enhance DNA unwinding, with a particular emphasis on the hydrophobic wedge in the Tnp domain. Our structures also show that the C nucleotide in the CT core motif is not recognized by the IS621 protein, while its complementary G is recognized by the side chain of N84, indicating that the preference of IS621 for the first core nucleotide could be altered by protein/bRNA engineering. Such engineered IS621 variants would expand the scope of the bridge recombination system.

The past two decades have witnessed the discovery of numerous RNA-guided single-protein nucleases, including both RNA-guided DNA nucleases (*e.g.*, Cas9[21], Cas12[22], IscB[23], TnpB[23,24], and Fanzor[25]), and RNA-guided RNA nucleases (*e.g.*, Argonaute[26] and Cas13[27]). In addition,

RNA-guided multi-protein complexes, such as CRISPR-associated transposases[28,29] and the CRISPR nuclease-protease[30,31], employ distinct protein subunits to perform RNA-mediated nucleic-acid recognition and enzymatic activities. DNA-targeting CRISPR enzymes, such as Cas9 and Cas12, recognize specific DNA sequences (called protospacer adjacent motifs) to induce local base-flipping upstream of a target sequence, thereby initiating guide–target base-pairing[32,33]. In contrast, IS621 uses the hydrophobic wedge to destabilize DNA duplexes adjacent to target sequences (LT/RT and LD/RD), thereby facilitating DNA unwinding and RNA–DNA hybridization. Our discovery of the IS110 family recombinases highlights the potential of diverse RNA-guided enzymes.

Supplementary Video 1 | Structure of the IS621 synaptic complex in the pre-strand exchange state.

The catalytic residues (S241 in the Tnp domain and D11/E60/D102/D105 in the RuvC domain) are shown as stick models. Disordered regions in the dDNA and tDNA are indicated by dotted lines.

Supplementary Video 2 | Structures of the RuvC/Tnp composite active sites.

The catalytic residues (S241 in the Tnp domain and D11/E60/D102/D105 in the RuvC domain) are shown as stick models. The Mg^{2+} ions are depicted as spheres. Disordered regions in the dDNA and tDNA are indicated by dotted lines.

Supplementary Video 3 | Structures of the IS621 synaptic complexes in the pre- and post-strand exchange states.

The three synaptic complex structures are superimposed based on the IS621.2 protomers to highlight their structural differences. Disordered regions in the dDNA and tDNA are indicated by dotted lines.

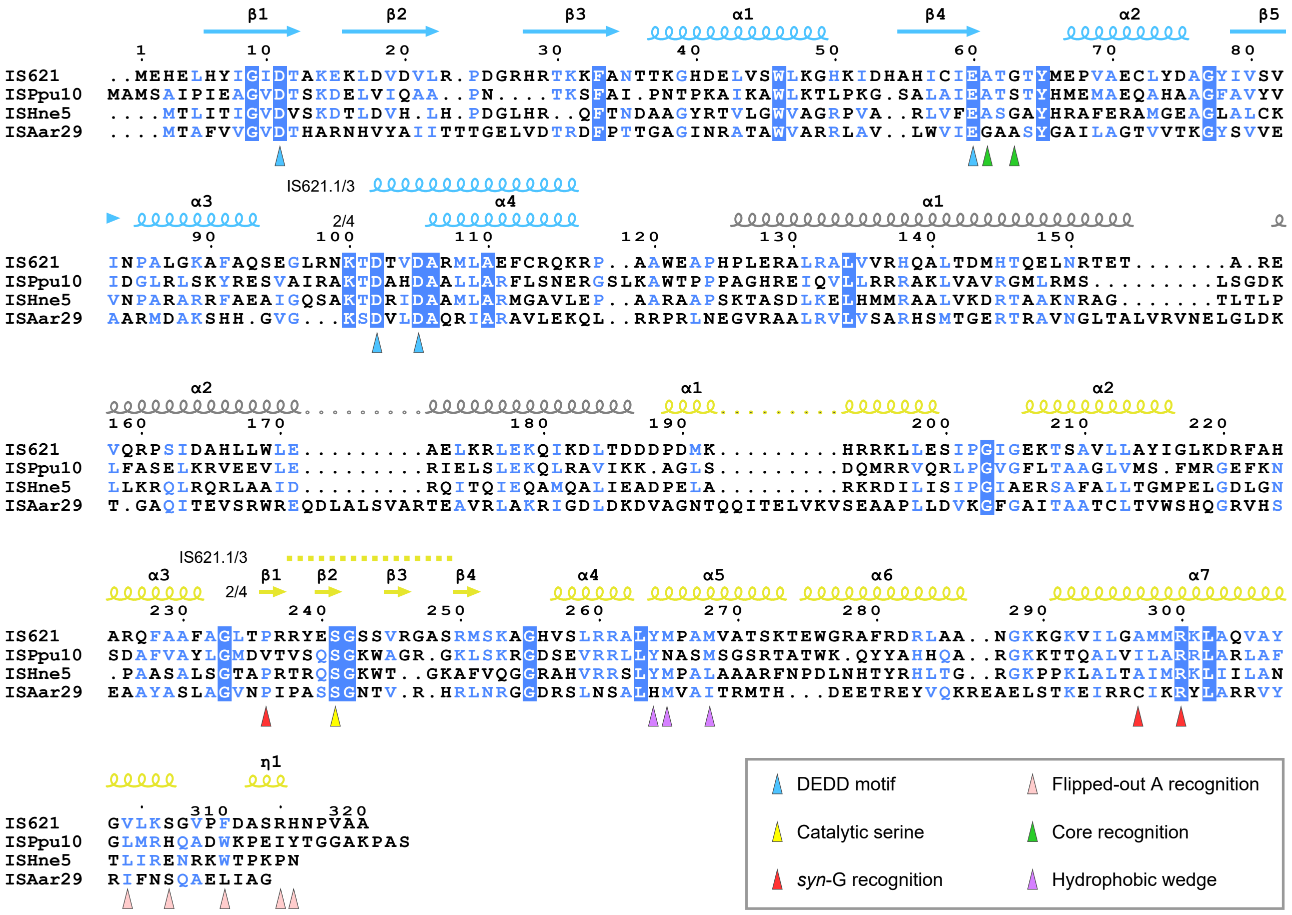

Supplementary Fig. 1 | Multiple sequence alignment of the IS110 family recombinases.

The figure was prepared using Clustal Omega (http://www.ebi.ac.uk/Tools/msa/clustalo) and ESPript3 (http://espript.ibcp.fr/ESPript/ESPript). Key residues are indicated by triangles.

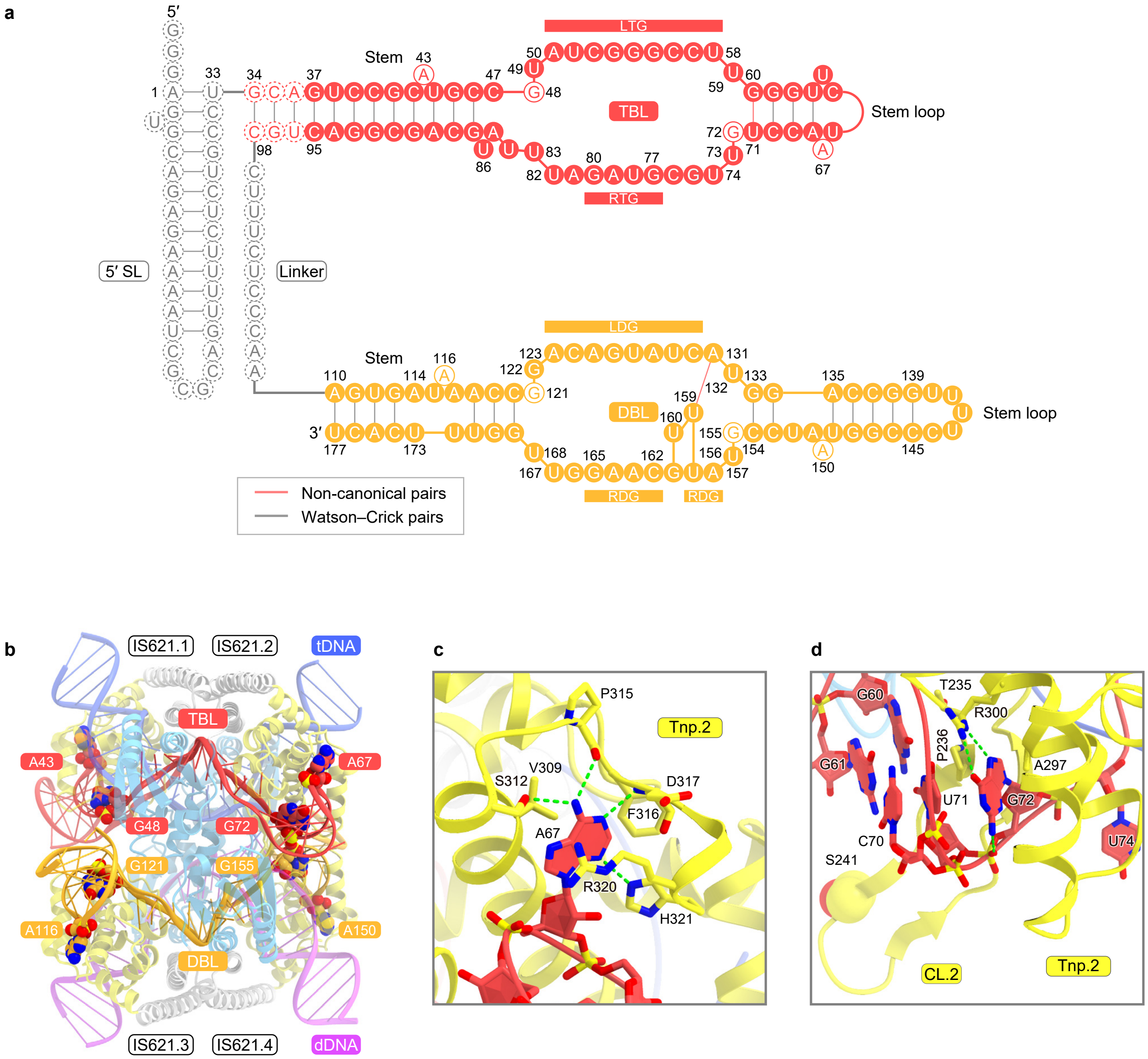

Supplementary Fig. 2 | Bridge RNA architecture.

a, Schematic of the bRNA. Disordered regions are indicated by dotted lines.

b, Common structural features in the TBL and DBL.

c,**d**, Recognition of A67 (**c**) and G72 (**d**) by the Tnp domain.

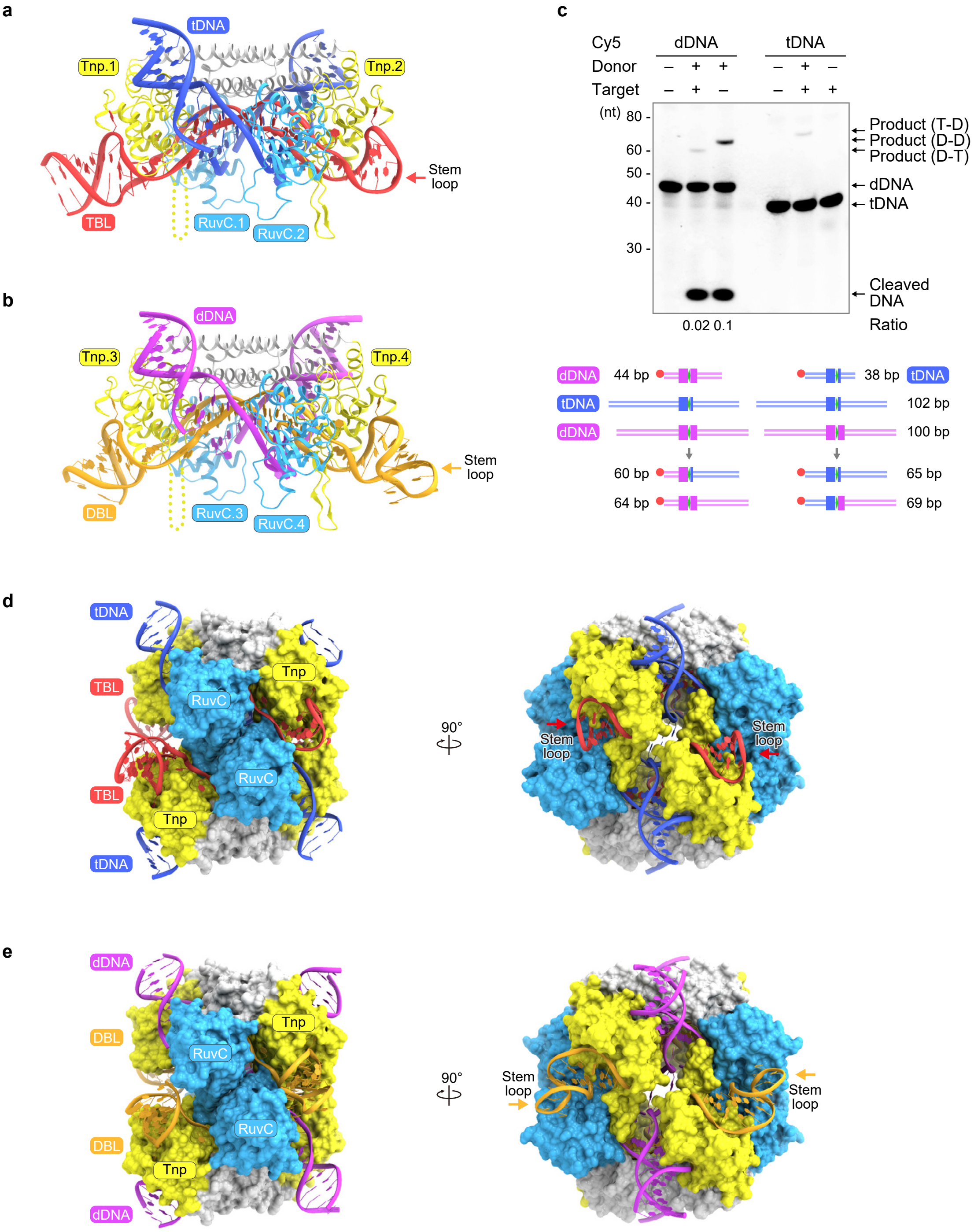

Supplementary Fig. 3 | Synaptic complex formation.

a,**b**, Structures of the IS621–TBL–tDNA (**a**) and IS621–DBL–dDNA (**b**) dimeric complexes.

c, *In vitro* DNA recombination experiments. The tDNA (38 bp) and dDNA (44 bp) substrates were labeled with Cy5 at the 5′ end of the top strand. The Cy5-tDNA (38 bp) or Cy5-dDNA (44 bp) was mixed with the non-labeled tDNA (102 bp) or dDNA (100 bp), and incubated with the IS621–bRNA complex at 37°C for 1 h. The reaction was then analyzed using an 18% TBE–urea gel. Recombination between the Cy5-dDNA and tDNA and between the Cy5-dDNA and dDNA yields 60- and 64-bp Cy5-labeled products, respectively. In contrast, recombination between the Cy5-tDNA and tDNA and between the Cy5-tDNA and dDNA yields 65- and 69-bp Cy5-labeled products, respectively. For the results with the labeled dDNA, the band intensities of the product and cleaved DNAs were quantified, and the recombination ratios (product DNA / product DNA + cleaved DNA) were calculated. Experiments were repeated at least three times with similar results.

d,**e**, Models of the IS621–TBL–tDNA (**d**) and IS621–DBL–dDNA (**e**) tetrameric complexes. The models were generated by superimposing IS621–TBL–tDNA and IS621–DBL–dDNA onto IS621–DBL–dDNA and IS621–TBL–tDNA in the synaptic complex.

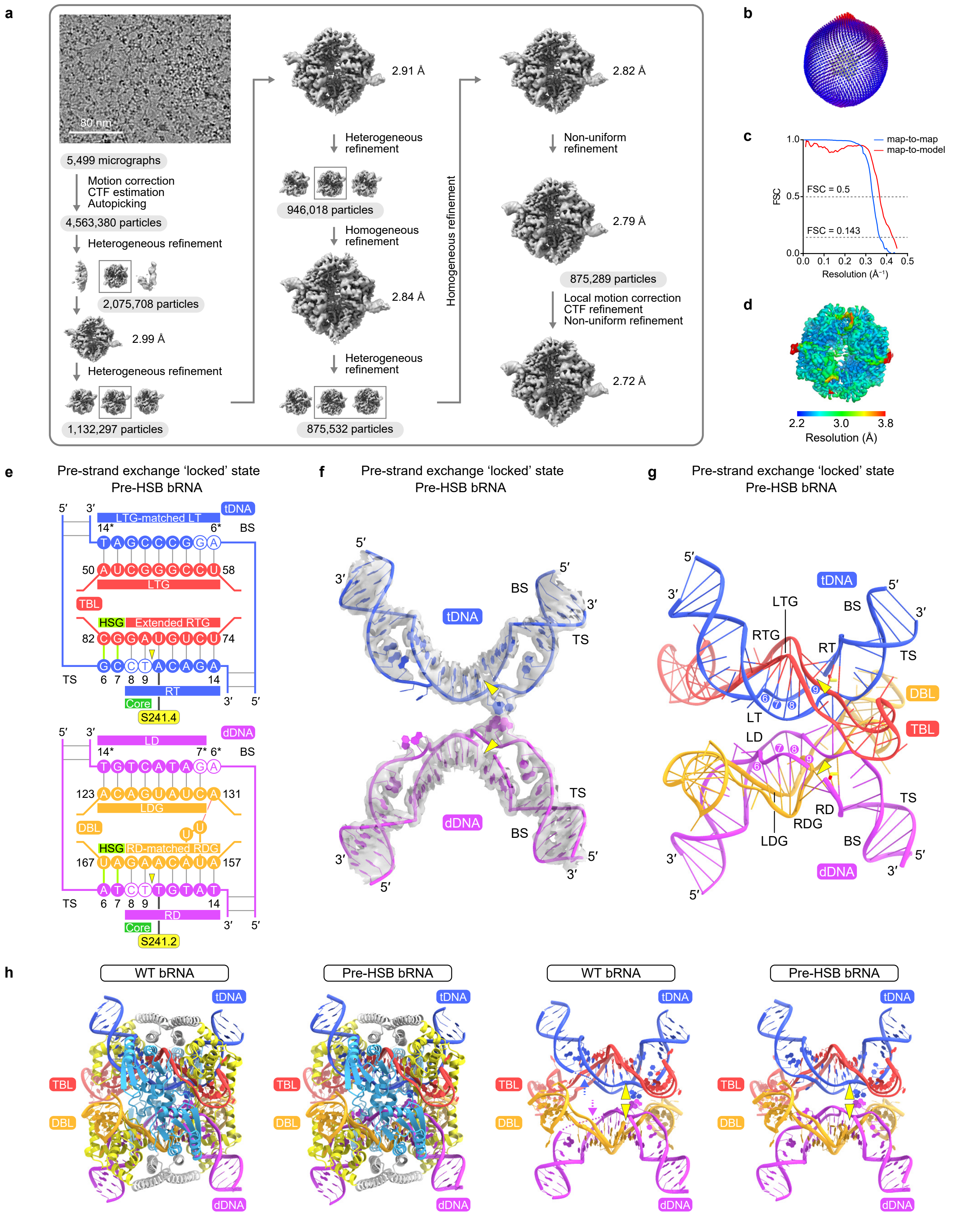

Supplementary Fig. 4 | Cryo-EM analysis of the IS621 synaptic complex with the pre-HSB bRNA in the pre-strand exchange 'locked' state.

a, Single-particle cryo-EM image processing workflow.

b, Angular distribution of particles in the final reconstruction.

c, FSC curves.

d, Cryo-EM density map, colored according to the local resolution.

e, Schematic of TBL–tDNA/DBL–dDNA. The base-pairs between the HSGs and DNA, which contribute to locking the synaptic complex in the pre-strand exchange state, are highlighted by green lines.

f, Cryo-EM density map for tDNA/dDNA.

g, Structure of TBL–tDNA/DBL–dDNA.

h, Structural comparison between the IS621 synaptic complexes with the WT bRNA and the pre-HSB bRNA in the pre-strand exchange states. DNA cleavage sites are indicated by yellow triangles. Disordered regions in the tDNA and dDNA are depicted as dotted lines and indicated by arrows.

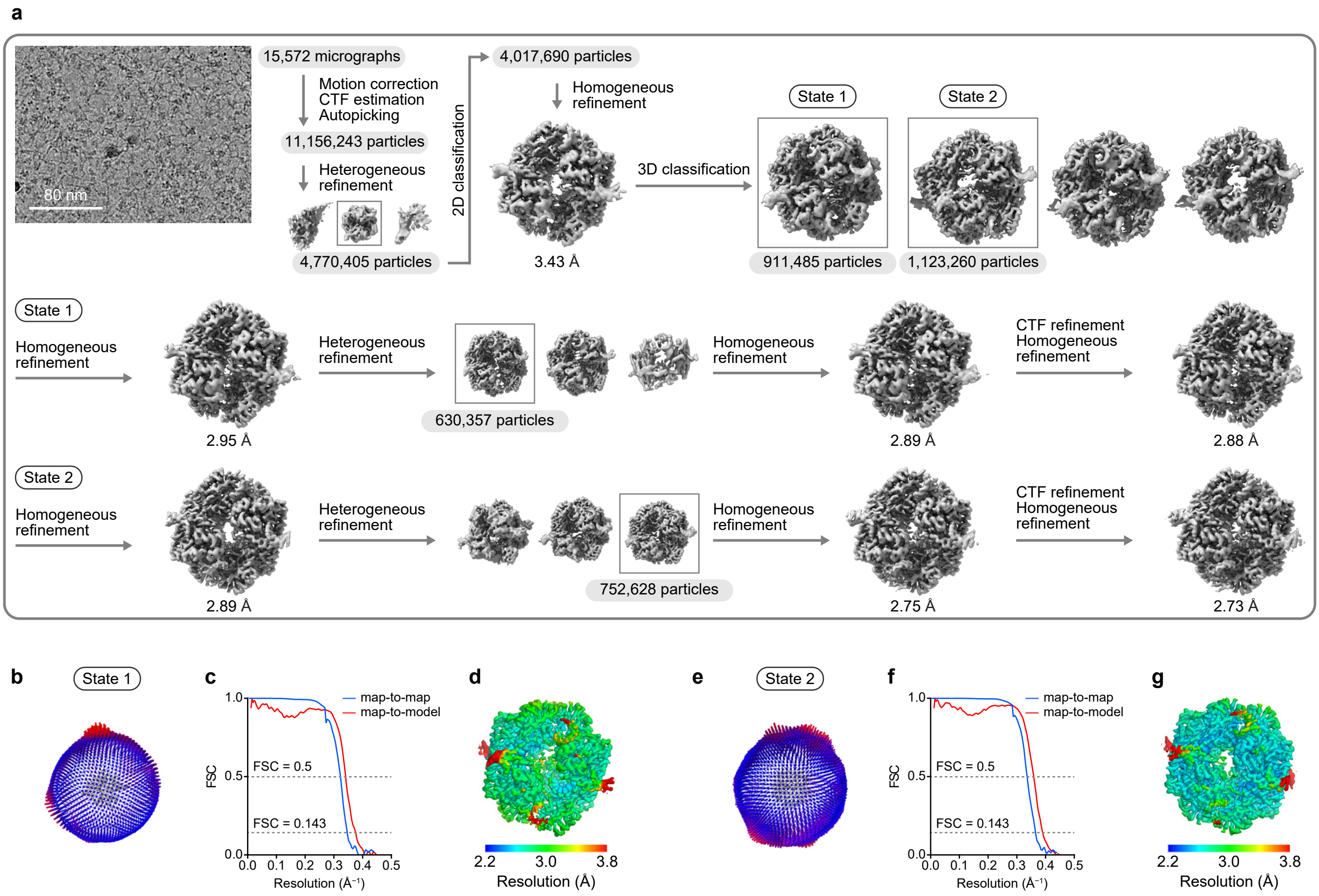

Supplementary Fig. 5 | Cryo-EM analysis of the IS621 synaptic complexes in the post-strand exchange states.
a, Single-particle cryo-EM image processing workflow.
b,**e**, Angular distributions of particles in the final reconstructions of State 1 (**b**) and State 2 (**e**).
c,**f**, FSC curves of State 1 (**c**) and State 2 (**f**).
d,**g**, Cryo-EM density maps of State 1 (**d**) and State 2 (**g**), colored according to the local resolution.

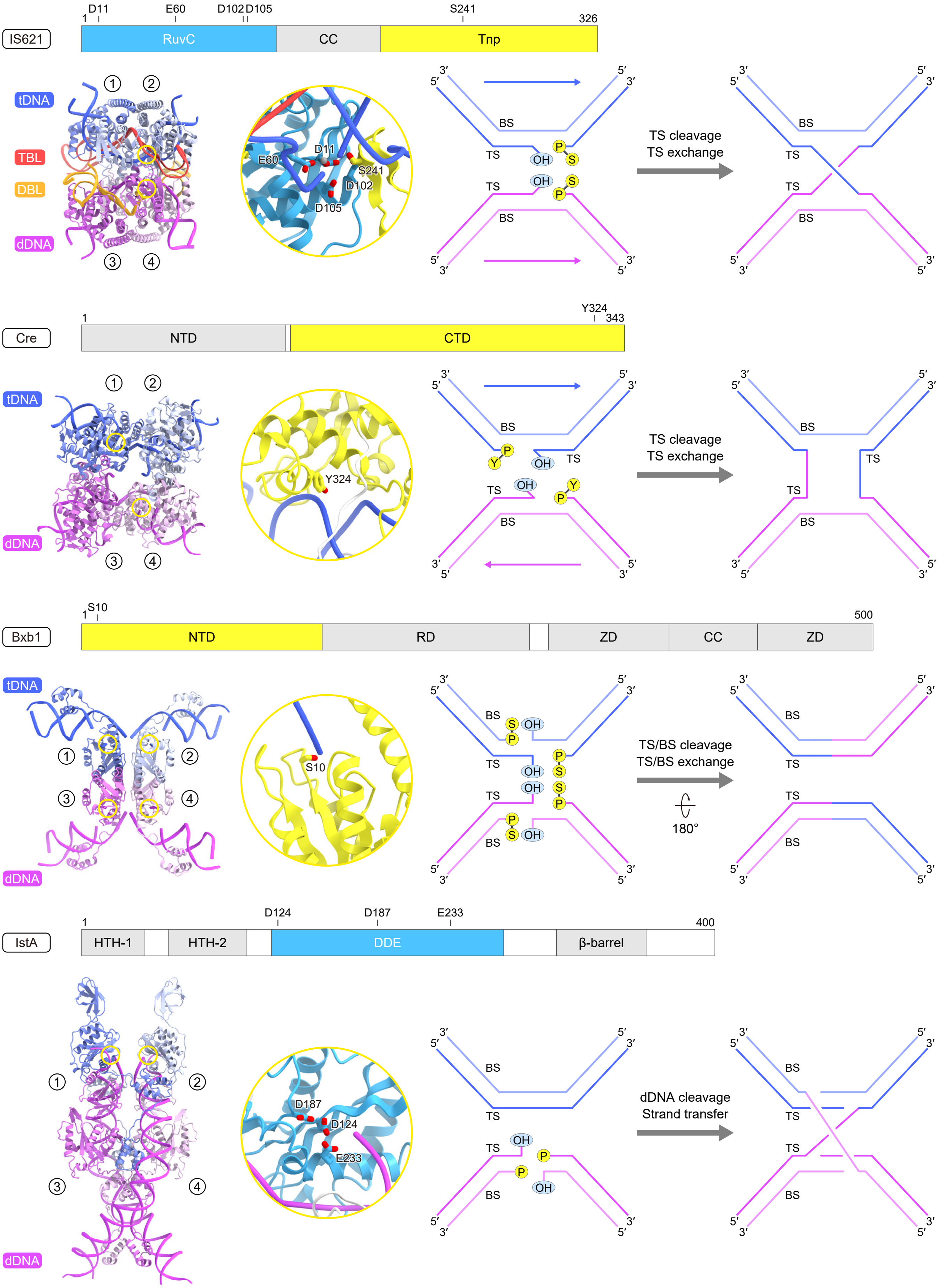
IS621
1
D11
E60
D102 D105
S241
326
RuvC
CC
Tnp
tDNA
TBL
DBL
dDNA
D11
E60
S241
D102
D105
TS cleavage
TS exchange
BS
TS
OH
P
S
Cre
1
Y324
343
NTD
CTD
Y324
Y
Bxb1
S10
500
RD
ZD
S10
TS/BS cleavage
TS/BS exchange
180°
IstA
D124
D187
E233
400
HTH-1
HTH-2
DDE
β-barrel
dDNA cleavage
Strand transfer
3′
5′

Supplementary Fig. 6 | Comparison of IS621 with other recombinases and transposases.

Comparison of the primary and tertiary structures and the DNA recombination mechanisms among the DEDD recombinase IS621, the tyrosine recombinase Cre (PDB: 1CRX), the serine recombinase Bxb1, and the DDE transposase IstA (PDB: 8B4H). The crystal structure of the γδ resolvase (PDB: 1ZR2) is shown as the representative structure of a serine recombinase, since no structural information is available for Bxb1. For comparison, the two DNA molecules bound to Cre and the γδ resolvase are labeled as tDNA and dDNA and colored blue and magenta, respectively, although they catalyse recombination between two DNA molecules with the identical recognition sequences (*lox*P for Cre and *res* for the γδ resolvase). For IS621 and Cre, the orientations of the two DNA molecules are indicated by arrows. The IstA structure binds a donor DNA (the inverted terminal repeats of the IS21 element), but lacks a target DNA. The four protomers in the synaptic complexes are numbered, and the active sites are marked by yellow circles. NTD, N-terminal domain; CTD, C-terminal domain; RD, recombinase domain; ZD, zinc ribbon domain; HTH, helix-turn-helix. TS, top strand; BS, bottom strand.

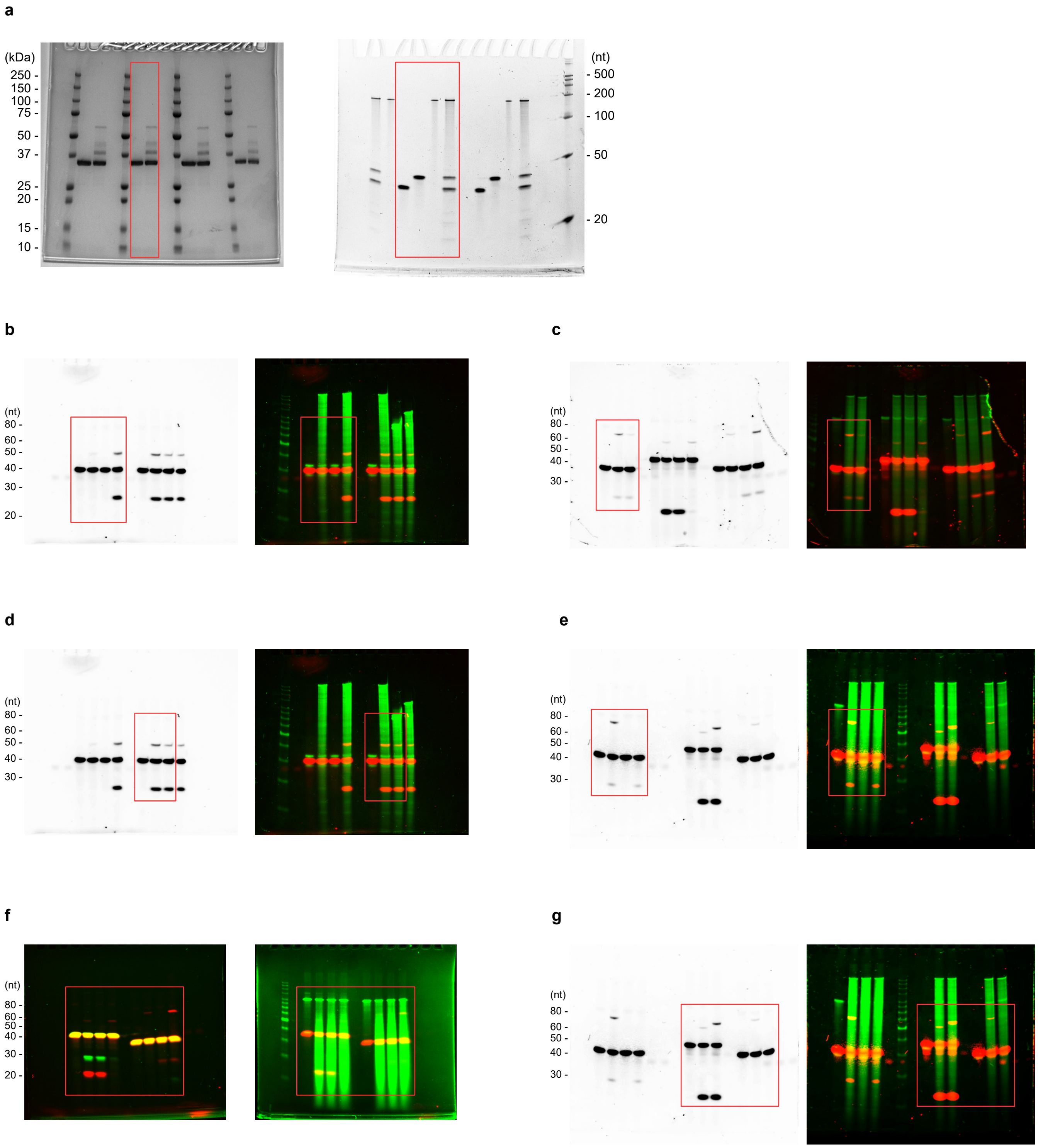

Supplementary Fig. 7 | Uncropped images of gels.

a–**g**, Uncropped gels used for Extended Data Fig. 2a (**a**), Extended Data Fig. 2b (**b**), Extended Data Fig. 6d (**c**), Extended Data Fig. 6e (**d**), Extended Data Fig. 6g (**e**), Extended Data Fig. 8b (**f**), and Supplementary Fig. 3c (**g**).

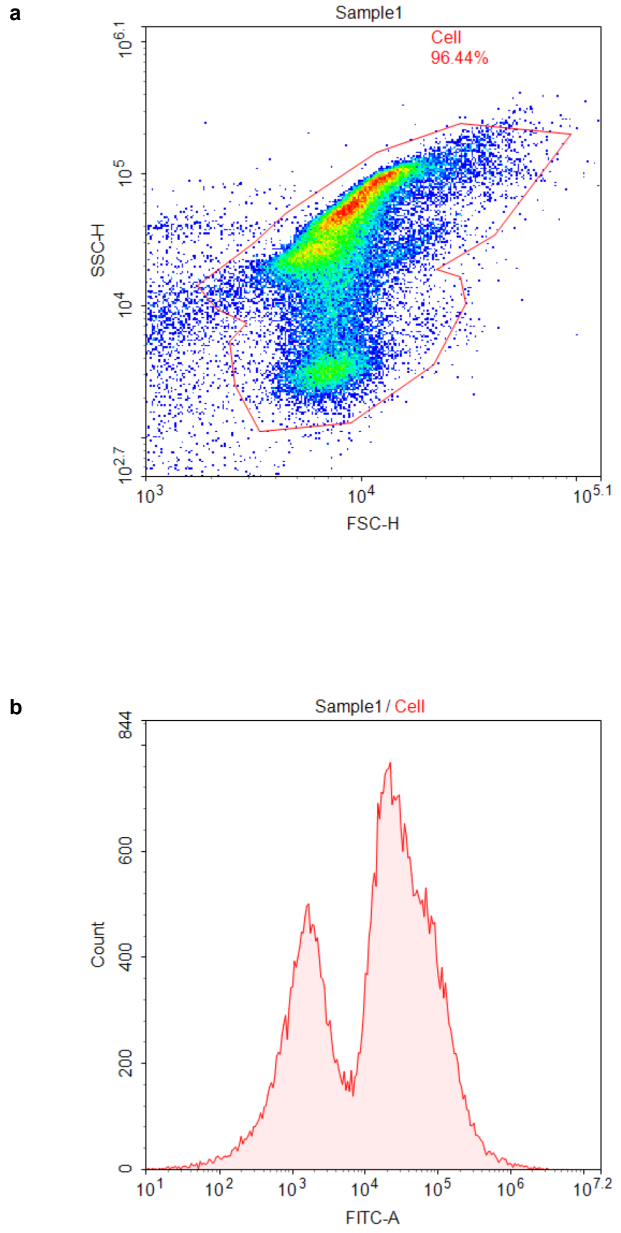

Supplementary Fig. 8 | Example of flow cytometry gating strategy.

a, Gating on FSC/SSC of cells.

b, Resulting histogram of FITC-A after gating in (**a**). The mean fluorescence intensity (MFI) of the lower plot was recorded for all experiments.
