# Supplementary material for: Structural mechanism of bridge RNA-guided recombination
Source: Nature. 2024 Jun 26;630(8018):994–1002. doi: 10.1038/s41586-024-07570-2 (PMC11208158; doi:10.1038/s41586-024-07570-2)
Supplement: Supplementary file 2 — Reporting Summary [file 41586_2024_7570_MOESM2_ESM.pdf]

nature portfolio

Corresponding author(s): Hiroshi Nishimasu, Patrick D. Hsu

Last updated by author(s): Apr 30, 2024

Reporting Summary

Nature Portfolio wishes to improve the reproducibility of the work that we publish. This form provides structure for consistency and transparency in reporting. For further information on Nature Portfolio policies, see our Editorial Policies and the Editorial Policy Checklist.

Statistics

For all statistical analyses, confirm that the following items are present in the figure legend, table legend, main text, or Methods section.

n/a	Confirmed	
☒	☐	The exact sample size (*n*) for each experimental group/condition, given as a discrete number and unit of measurement
☐	☒	A statement on whether measurements were taken from distinct samples or whether the same sample was measured repeatedly
☒	☐	The statistical test(s) used AND whether they are one- or two-sided<br>*Only common tests should be described solely by name; describe more complex techniques in the Methods section.*
☒	☐	A description of all covariates tested
☒	☐	A description of any assumptions or corrections, such as tests of normality and adjustment for multiple comparisons
☐	☒	A full description of the statistical parameters including central tendency (e.g. means) or other basic estimates (e.g. regression coefficient) AND variation (e.g. standard deviation) or associated estimates of uncertainty (e.g. confidence intervals)
☒	☐	For null hypothesis testing, the test statistic (e.g. *F*, *t*, *r*) with confidence intervals, effect sizes, degrees of freedom and *P* value noted<br>*Give P values as exact values whenever suitable.*
☒	☐	For Bayesian analysis, information on the choice of priors and Markov chain Monte Carlo settings
☒	☐	For hierarchical and complex designs, identification of the appropriate level for tests and full reporting of outcomes
☒	☐	Estimates of effect sizes (e.g. Cohen's *d*, Pearson's *r*), indicating how they were calculated

Our web collection on statistics for biologists contains articles on many of the points above.

Software and code

Policy information about availability of computer code

Data collection	EPF v3.2.0.4776
Data analysis	cryoSPARC v4.4.0, Alphafold2, COOT v0.9.8.93, Phenix v1.20.1, Servalcat v0.4.65, UCSF Chimera X v1.4, CueMol2 (http://www.cuemol.org/), GraphPad Prism 10

For manuscripts utilizing custom algorithms or software that are central to the research but not yet described in published literature, software must be made available to editors and reviewers. We strongly encourage code deposition in a community repository (e.g. GitHub). See the Nature Portfolio guidelines for submitting code & software for further information.

Data

Policy information about availability of data

All manuscripts must include a data availability statement. This statement should provide the following information, where applicable:

- Accession codes, unique identifiers, or web links for publicly available datasets
- A description of any restrictions on data availability
- For clinical datasets or third party data, please ensure that the statement adheres to our policy

Cryo-EM density maps have been deposited in the Electron Microscopy Data Bank under the accession codes EMD-37827 (pre-strand exchange state), EMD-37828 (pre-strand exchange locked state), EMD-37829 (HJ intermediate state), and EMD-37830 (HJ resolution state). Atomic coordinates have been deposited in the

Protein Data Bank under IDs 8WT6 (pre-strand exchange state), 8WT7 (pre-strand exchange locked state), 8WT8 (HJ intermediate state), and 8WT9 (HJ resolution state). PDB: 7S4X, 1CRX, 3CRX, 1ZR2, and 8B4H were used for structural comparison.

Research involving human participants, their data, or biological material

Policy information about studies with human participants or human data. See also policy information about sex, gender (identity/presentation), and sexual orientation and race, ethnicity and racism.

Reporting on sex and gender	n/a
Reporting on race, ethnicity, or other socially relevant groupings	n/a
Population characteristics	n/a
Recruitment	n/a
Ethics oversight	n/a

Note that full information on the approval of the study protocol must also be provided in the manuscript.

Field-specific reporting

Please select the one below that is the best fit for your research. If you are not sure, read the appropriate sections before making your selection.

☒ Life sciences ☐ Behavioural & social sciences ☐ Ecological, evolutionary & environmental sciences

For a reference copy of the document with all sections, see nature.com/documents/nr-reporting-summary-flat.pdf

Life sciences study design

All studies must disclose on these points even when the disclosure is negative.

Sample size	No sample size calculations were performed, as this is generally not relevant to biochemical and bacterial experiments.
Data exclusions	Damaged particles were excluded during 2D and 3D classification in cryo-EM reconstitution to obtain high-resolution structures, according to the standard procedures in single-particle cryo-EM analysis.
Replication	All functional experiments were performed with independent replicates, according to widely adopted protocols in the field (three technical or biological replicates). Biochemical and bacterial experiments were performed with at least three technical and biological replicates, respectively.
Randomization	No randomization was performed. Randomization is not relevant to this study, as human subjects or live animals were not involved.
Blinding	No blinding was performed. Blinding is not relevant to this study, as human subjects or live animals were not involved.

Reporting for specific materials, systems and methods

We require information from authors about some types of materials, experimental systems and methods used in many studies. Here, indicate whether each material, system or method listed is relevant to your study. If you are not sure if a list item applies to your research, read the appropriate section before selecting a response.

Materials & experimental systems

n/a	Involved in the study	
☒	☐	Antibodies
☐	☒	Eukaryotic cell lines
☒	☐	Palaeontology and archaeology
☒	☐	Animals and other organisms
☒	☐	Clinical data
☒	☐	Dual use research of concern
☒	☐	Plants

Methods

n/a	Involved in the study	
☒	☐	ChIP-seq
☐	☒	Flow cytometry
☒	☐	MRI-based neuroimaging

Eukaryotic cell lines

Policy information about cell lines and Sex and Gender in Research

Cell line source(s)	Thermo Fisher Scientific
Authentication	*Describe the authentication procedures for each cell line used OR declare that none of the cell lines used were authenticated.*
Mycoplasma contamination	*Confirm that all cell lines tested negative for mycoplasma contamination OR describe the results of the testing for mycoplasma contamination OR declare that the cell lines were not tested for mycoplasma contamination.*
Commonly misidentified lines (See ICLAC register)	*Name any commonly misidentified cell lines used in the study and provide a rationale for their use.*

Plants

Seed stocks	not relevant
Novel plant genotypes	not relevant
Authentication	not relevant

Flow Cytometry

Plots

Confirm that:

- [x] The axis labels state the marker and fluorochrome used (e.g. CD4-FITC).
- [x] The axis scales are clearly visible. Include numbers along axes only for bottom left plot of group (a 'group' is an analysis of identical markers).
- [ ] All plots are contour plots with outliers or pseudocolor plots.
- [x] A numerical value for number of cells or percentage (with statistics) is provided.

Methodology

Sample preparation	All cells were E. coli. Cells were scraped from agar plates, resuspended in DPBS, and diluted to the appropriate concentration.
Instrument	Agilent NovoCyte Quanteon
Software	NovoExpress software was used for flow cytometry analysis
Cell population abundance	At least 50,000 cells were analyzed.
Gating strategy	The majority (95%) of cells were gated on FSC/SSC. No further gating was performed.

- [x] Tick this box to confirm that a figure exemplifying the gating strategy is provided in the Supplementary Information.
